# Oscillations in the prefrontal-hippocampal circuit couple to respiration-related oscillations during all phases of a working memory task

**DOI:** 10.3389/fnbeh.2025.1669111

**Published:** 2025-10-30

**Authors:** Sunandha Srikanth, Dylan Le, Yudi Hu, Jill K. Leutgeb, Stefan Leutgeb

**Affiliations:** ^1^Department of Neurobiology, School of Biological Sciences, University of California San Diego, La Jolla, CA, United States; ^2^Kavli Institute for Brain and Mind, University of California San Diego, La Jolla, CA, United States

**Keywords:** respiration-related oscillations, theta oscillations, hippocampus, prefrontal cortex, local field potentials, odor-guided memory task

## Abstract

Oscillatory activity is thought to coordinate neural computations across brain regions, and theta oscillations are critical for learning and memory. Because respiration-related oscillations (RROs) in rodents can be identified in the prefrontal cortex (PFC) and the hippocampus in addition to canonical theta oscillations, we asked whether odor-cued working memory may be supported by both of these two oscillations. We first confirmed that RROs were propagated to the hippocampus and PFC and that RRO frequency spans a broad range that partially overlaps with canonical theta frequency. During all task phases, we found coherence between PFC and hippocampus at the RRO frequency, irrespective of whether RROs and canonical theta oscillations overlapped or differed in frequency. In parallel, there was also high coherence across PFC and hippocampus at theta frequency, except that the coupling at theta was weakest during odor sampling. Therefore, long-range coordination between brain regions occurs at more than one oscillation frequency in a working memory task, but the two types of oscillations did not show evidence of conjunctively supporting working memory.

## Introduction

Brain oscillations are thought to coordinate neural computations across cortical and sub-cortical brain regions by synchronizing network activity (Buzsaki and Draguhn, 2004). For example, coordination in the theta frequency range (6–12 Hz) is prominent in neural circuits that support memory function, and accordingly, theta oscillations are not only found in the hippocampus, but also in directly and indirectly connected brain regions (Buzsaki, 2002; Colgin, 2011; Backus et al., 2016). Importantly, coordinated oscillations across brain regions are not only matching in frequency, but analyses of local field potential (LFP) recordings also reveals that oscillation patterns can be highly coherent [e.g., between hippocampus and medial prefrontal cortex (mPFC)]. In addition, neuronal firing patterns of many prefrontal neurons are phase-locked to the hippocampal theta rhythm (Hyman et al., 2005; Jones and Wilson, 2005; Siapas et al., 2005; Zielinski et al., 2019). Since oscillations within a network correspond to rhythmic fluctuations in excitability, the synchronized oscillations across brain regions allow for windows of peak excitability that enable efficient communication between brain regions (Fries, 2005). Accordingly, the accuracy of spatial coding in hippocampus and mPFC has been reported to be coupled on a cycle-by-cycle basis (Zielinski et al., 2019). Furthermore, prefrontal-hippocampal oscillatory strength correlates with performance in spatial working memory tasks in rodents (Jones and Wilson, 2005; Benchenane et al., 2010; Zielinski et al., 2019), which suggests that oscillatory coupling supports memory function and raises the question whether an even broader network is dynamically synchronized during task performance.

Along with the canonical theta oscillations that are most prominent in the hippocampus, oscillations that are related to the respiration rhythm and encompass an overlapping frequency range (3–12 Hz) have been described (Rojas-Libano et al., 2014; Yanovsky et al., 2014; Lockmann et al., 2016; Nguyen Chi et al., 2016; Tort et al., 2025). Respiration is paced by brainstem breathing centers (Feldman et al., 2013), and the nasal airflow that is generated by breathing then activates olfactory sensory neurons in the nasal epithelium during each breathing cycle (Wu et al., 2017). This mechanism entrains local oscillatory activity in the olfactory bulb (OB), and the respiratory rhythm and OB oscillations are thus tightly coupled. In particular, a causal role of nasal airflow for olfactory oscillations has been established by the finding that the entrainment of OB network activity is diminished when nasal airflow is restricted by means of naris occlusion or tracheal breathing (Onoda and Mori, 1980; Phillips et al., 2012).

Respiration-entrained activity of OB neurons is transmitted to olfactory-associated cortical areas such as the piriform cortex (Fontanini et al., 2003) and the barrel cortex (Ito et al., 2014), but also to more indirectly connected subcortical and cortical regions across the brain, including the medial prefrontal cortex (mPFC) (Biskamp et al., 2017) and the hippocampus (Yanovsky et al., 2014; Lockmann et al., 2016; Nguyen Chi et al., 2016; Tort et al., 2018). Throughout these brain regions, respiration-related oscillations (RROs) can be distinguished from other types of oscillations by confirming the coupling to either the respiration rhythm and/or olfactory bulb oscillations. Consistent with the definition of RROs as respiration or OB-oscillation related, these oscillatory patterns in the mPFC, barrel cortex and the hippocampus are disrupted when manipulating nasal airflow or signals from the OB (Ito et al., 2014; Yanovsky et al., 2014; Nguyen Chi et al., 2016; Biskamp et al., 2017; Moberly et al., 2018).

Because the overlap in frequency between RROs and canonical theta can be confounding for separately analyzing these oscillation patterns, characterization of RROs has mostly focused on periods when RROs differ in frequency from theta oscillations during running, immobility and anesthesia (Yanovsky et al., 2014; Nguyen Chi et al., 2016). In these analyses, oscillations at the respiratory frequency have a different depth profile than theta oscillations in the hippocampus, which supports the notion that RROs are separately generated oscillations that co-occur with theta oscillations in the hippocampus. In contrast, there is also evidence that olfactory oscillations and hippocampal theta oscillations become coherent during periods of sniffing in odor learning and discrimination tasks (Macrides et al., 1982; Kay, 2005). These latter studies suggest that the coherence between hippocampal and olfactory networks mediates sensorimotor integration in the hippocampus. A possible source for the conflicting reports of either parallel oscillations or the coupling of RROs and hippocampal oscillations is that these reports have not considered the existence of two types of theta oscillations in the hippocampus – movement-related theta oscillations and sensory-evoked theta oscillations (Vanderwolf, 1969; Kramis et al., 1975) or the possibility that RROs and theta co-exist as distinct oscillations. We therefore investigated whether coherence of RROs and of theta oscillations across brain regions independently or jointly vary across phases within a memory task. Furthermore, we reasoned that coupling at the RRO frequency may be particularly pronounced when olfactory cues that are relevant for memory performance are processed. We therefore performed recordings in an odor-cued hippocampus-dependent working memory task. To be able to identify RROs and theta oscillations throughout the behavior, we recorded OB oscillations simultaneously with hippocampal oscillations. Furthermore, we also simultaneously recorded from mPFC to examine whether the convergence of RROs and hippocampus-coupled theta in mPFC would allow for dynamic coupling of each of these two types of oscillations, which could in turn serve as a conduit for coordinating memory and sensory processing in the prefrontal-hippocampal circuit.

## Materials and Methods

### Subjects

Eight mice [VGAT-cre 129S6(FVB)-Slc32a1^tm2(cre)Lowl^/MwarJ, Jackson Labs; *n* = 4 male, *n* = 4 female] that were 4 months old and weighed 20–30 g were used as subjects in the odor-cued working memory task. The sample size was determined based on the number of mice used in previous studies with recordings of RROs in awake behaving mice. An additional 4 mice of the same line were used for joint respiratory rhythm and OB recordings. All mice were single-housed in a reverse 12 h dark/light cycle (lights off at 8 am). Mice were restricted to 85%–90% of their *ad libitum* weight and given full access to water. All the training and testing was performed during the dark phase. All procedures were conducted in accordance with the University of California, San Diego Institutional Animal Care and Use Committee.

### Surgery

Mice that were prepared for recordings in the working memory task were anesthetized with isoflurane (induction: 3%, maintenance: 1.5%–2%) and mounted in a stereotaxic frame (David Kopf Instruments, Model 1900). The scalp was cleaned and retracted using a midline incision, and the skull was leveled between bregma and lambda. Five holes were drilled in the skull to attach anchor screws. A hole was drilled above the cerebellum to place a ground screw. Craniotomies were performed over four brain regions on the right hemisphere [OB: +4.2 mm anteroposterior (A/P), 0.6 mm mediolateral (M/L); mPFC: +1.8 to 2 mm A/P, 0.4 mm M/L; dorsal hippocampus (dHC): −1.9 mm A/P, 2.0 mm M/L; ventral hippocampus (vHC): −3.3 mm A/P, 3.5 mm M/L], and dura was removed. Wires were implanted in the four brain regions [OB: −1.2 mm dorsoventral (D/V); mPFC: −1.4 mm D/V; dHC: −1.8 mm D/V; vHC: −3.5 mm D/V] to record LFPs. The wires were threaded through a circuit board with a connector, and the implant was secured with dental cement. Postoperative care was administered as needed, and mice were allowed to recover for a minimum of 5 days before training them on the behavioral task.

To confirm that OB oscillations provide a reliable estimate of breathing frequency across the analyzed range (3–12 Hz), joint OB wire and thermocouple implantation was performed as per previously established protocols (McAfee et al., 2016) in 4 mice. Briefly, a thermocouple was placed in a hollow space above the nasal cavity (Nasal Fissure +3.1 mm) following a craniotomy. The thermocouple was held in place with dental cement. A second craniotomy was performed over the OB [+4.2 mm anteroposterior (A/P), 0.6 mm mediolateral (M/L)], and a wire was implanted in the OB [−1.2 mm dorsoventral (D/V)]. The thermocouple as well as the electrode wire were threaded through a circuit board with a connector. The implant was secured with dental cement. Postoperative care was administered, and mice were allowed to recover for a minimum of 5 days before recording.

### Histological procedures

Mice were perfused with 0.1 M phosphate-buffered saline (PBS) followed by 4% paraformaldehyde in PBS solution. Brains were post-fixed for 24 h in 4% paraformaldehyde and then cryoprotected in 30% sucrose solution for 2 days. Brains were then frozen and sliced into 40 μm coronal sections using a sliding microtome. Sections were mounted on electrostatic slides, stained with cresyl violet and coverslipped with Permount (Fisher Scientific, SP15500) to visualize recording locations. Slides were imaged using a virtual slide microscope (Olympus, VS120).

### Odor delivery

A custom plastic odor port was machined, and two IR LEDs (transmitter and receiver) were placed at the entrance of the odor port to detect nose pokes. These LEDs were connected to an Arduino board (Arduino Mega 2560) which was programmed to detect nose pokes and deliver an odor through a custom-made olfactometer. A hole was drilled at the bottom of the odor port to deliver the odor at a flow rate of up to 1 L/min. Two neutral odors (ethyl acetate and isoamyl acetate) were used in the task. These odors were freshly prepared daily in mineral oil (1:5 ratio by volume). One of the two odors was chosen using the rand function in MATLAB and delivered on each trial. A minimum interval of 2 s between odor deliveries was imposed to prevent triggering the odor delivery twice within a single trial. A custom written MATLAB script was used to deliver the odor as well as to send a TTL pulse to the Neuralynx acquisition system to timestamp the odor delivery, nose poke in and nose poke out. Precise timestamps for nose poke entry, odor delivery ON, odor delivery OFF and nose poke exit were recorded for four of the seven animals used in our analyses. For the other three animals, precise timestamps were recorded only for odor delivery ON and OFF.

### Behavior

Mice were trained on an odor-cued working memory task following the stereotaxic surgery. The room was dimly lit and stable environmental cues were placed. The task was performed on a figure-eight maze that was 50 cm above the ground, 75 cm long, and 50 cm wide with 5 cm wide runways. The custom-made olfactometer was placed on one end of the stem arm. The maze was cleaned with 70% alcohol after each animal used the maze. Animals were trained in phases. On the first day, animals were allowed to freely explore the maze for 10 min for habituation. After habituation for 1 day, animals started the first phase of training. In the first phase, animals were gently guided to the odor port to break an IR beam at the entrance of the odor port upon which an odor was delivered. Animals were required to sniff the odor for at least 1 s and run to the other end of the stem arm where they were forced to make the correct choice. They were then rewarded with a single chocolate sprinkle (Betty Crocker Parlor Perfect Chocolate Sprinkles) that was made available at the reward zone. Animals performed 60 trials per day. Once animals learned to nose poke in the odor port and run to the opposite end of the stem arm without guidance in all 60 trials on two consecutive days, they were ready for the second phase. In the second phase, animals performed the task without guidance to nose poke into the odor port and were given a choice to turn in either direction at the other end of the stem arm. Responses on all 60 trials were recorded and analyzed. The data from the second phase are reported in the Section “Results.” For this phase, we confirmed that the turn direction at the odor port was unrelated to the turn direction at the choice point (*n* = 1221 trials, χ^2^ = 0.27, *p* = 0.61) and that the path for upcoming right and left choices was centered on the stem up to ∼15 cm before the T junction. Therefore, our task can be considered to correspond to a version with olfactory working memory (Fujisawa et al., 2008; Fujisawa and Buzsaki, 2011) rather than a version in which the motor response is apparent immediately after odor sampling (Symanski et al., 2022).

### Electrophysiological recordings

Local field potentials were recorded using chronically implanted stainless steel wires that were insulated except at the tip. Implanted wires were connected to a head-mounted preamplifier and via a tether to a 32-channel digital data acquisition system (Neuralynx, Bozeman, MT). Continuous LFP was sampled at 32000 Hz and band-pass filtered between 0.1 and 1000 Hz. Position data of a red and a green LED located on either side of the head-mounted preamplifier were tracked by a video camera at a sampling frequency of 30 Hz to determine the spatial location of the animals while they performed the task.

### LFP analysis

Raw LFP signals were down-sampled to 2000 Hz and a Morlet wavelet of width ratio = 6 was used to determine the power and phase of the oscillations at 30 log-spaced frequencies in the 3–20 Hz range. An average spectrogram was constructed for each maze zone in each trial. As a measurement for coherence, we used intersite phase clustering (ISPC) (Cohen, 2014). For ISPC, phase differences between oscillations on pairs of recording sites are calculated for each frequency, and the length of the resultant vector of phase differences is measured. ISPC is similar to the commonly used spectral coherence (or magnitude-squared coherence) except that phase values are weighted by power values for spectral coherence, but not for ISPC. A coherence measurement that is entirely independent of spectral power is, because of the lower power of OB oscillations at higher respiration rates, preferred for our analyses.

Local field potential analyses were done separately for each maze zone (i.e., return arms, odor sampling, stem, reward zone). For analysis involving predominant frequencies within a region, a peak was detected in the 3–12 Hz range for RROs and in the 7–12 Hz range for movement-related and sensory-evoked theta oscillations. Peak values were calculated by finding the highest peak using the “findpeaks” function in MATLAB. If no peak was detected, that trial was omitted from analysis for that maze zone. To calculate shuffled coherence, 100 pairs of trials were selected at random. Then, for each of the 100 pairs, and for every pair of recording sites (say, X and Y) and for every maze zone, phase differences were calculated between phases corresponding to X in one trial and to Y in another trial. Coherence values were considered significant if they exceeded the 95th percentile of shuffled values. Cross Frequency Coupling analysis was performed as follows: LFP from the entire session was first bandpass filtered using two-way least-squares FIR filtering (“eegfilt” function in the EEGLAB toolbox in MATLAB). The LFP corresponding to the amplitude timeseries was filtered in the 10–100 Hz range in 5 Hz intervals and the LFP corresponding to the phase timeseries was filtered in the 2–20 Hz range in 1 Hz intervals. Filtered signals in each interval were then processed using a Hilbert transform, with the resultant analytical signal yielding the instantaneous phase angle and amplitude. Then, for each task phase, the corresponding segments from each trial were concatenated to form one long time series consisting only of phase or amplitude estimates from the corresponding task phase. A co-modulogram was computed for each task phase as previously described (Tort et al., 2010; Tavares and Tort, 2022).

### Statistics

All statistics were performed using built-in functions in MATLAB (R2019b). Non-parametric tests such as Kolmogorov-Smirnov (KS), Wilcoxon and Friedman tests were performed. In particular, Friedman tests were performed on the animal level with the sessions as repeated measures. Then, Wilcoxon tests were performed *post hoc*. Circular statistics was performed using the Circular Statistics Toolbox on MATLAB (Berens, 2009). Corrections for multiple tests were performed using the Holm-Bonferroni method to determine which comparisons were significant.

## Results

To investigate the coupling between RROs and canonical theta oscillations across brain regions, we simultaneously recorded LFP signals in OB, mPFC, and hippocampus. Because vHC is connected more strongly to mPFC than dHC (Hoover and Vertes, 2007), we placed separate recording electrodes in dHC and vHC. Within mPFC, we focused on the prelimbic, infralimbic, and anterior cingulate areas because of their direct and indirect connections with hippocampus. RROs as well as theta oscillations have been detected in all of these regions in previous studies (Biskamp et al., 2017; Tort et al., 2018).

**FIGURE 1:**
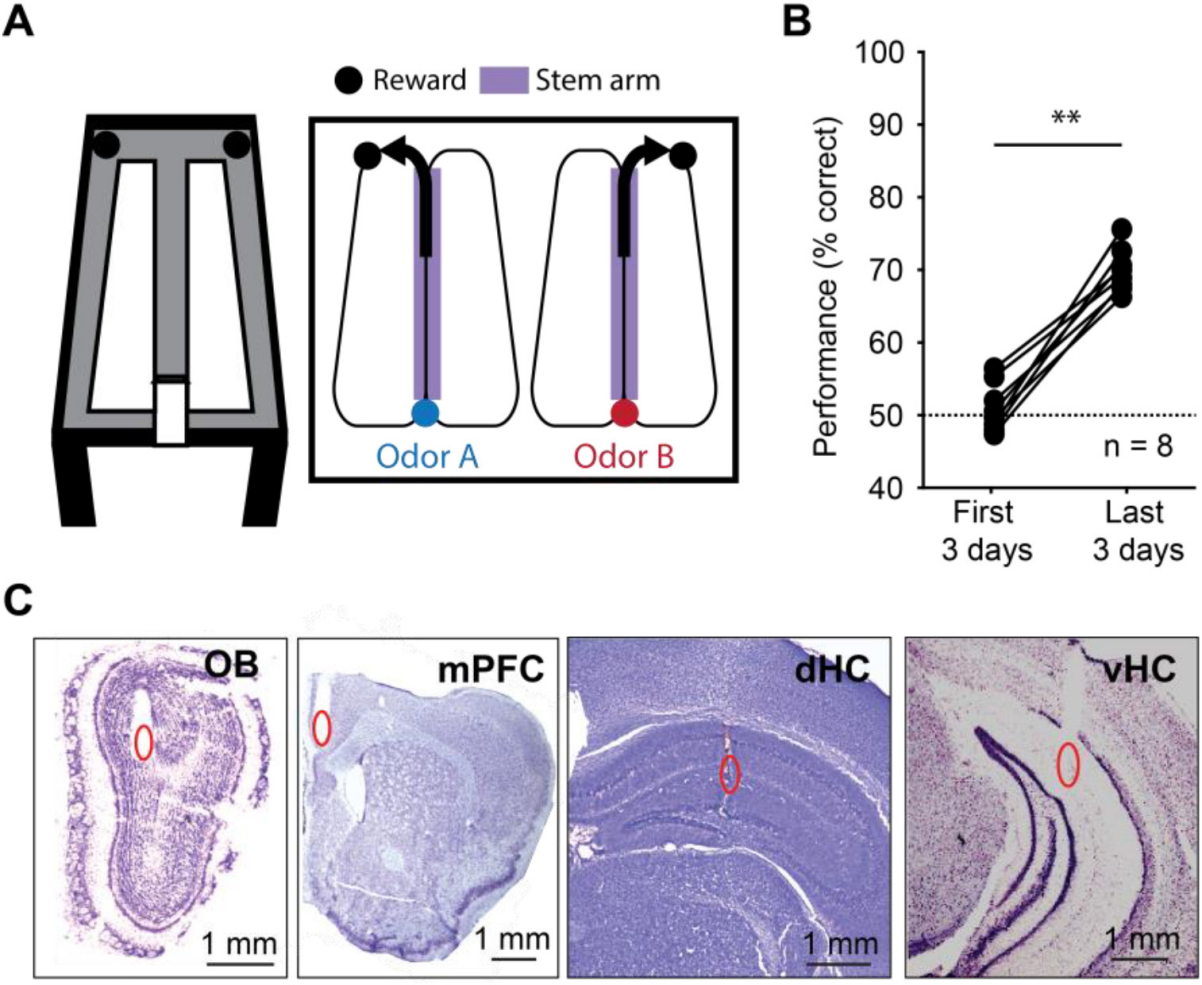
Mice performed an odor-cued working memory task with high accuracy. **(A)** Schematic of the odor-cued working memory task. Mice were trained to sniff one of two pseudo-randomly delivered odors at an odor port at the bottom of the stem arm and make a turn at the top of the stem arm based on the odor they sampled. The relation between odor identity and turn direction remained consistent for each mouse. A food reward was provided at the reward zones for correct choices, and mice returned to the odor port by running on the side arms. **(B)** Performance increased between the first 3 days and the last 3 days of behavioral testing (*n* = 8 mice, *Z* = 2.45, *p* = 0.007, one-tailed Wilcoxon signed-rank test). Dashed line, chance level. **(C)** Example recording electrode locations in the olfactory bulb (OB), medial prefrontal cortex (mPFC), dorsal hippocampus (dHC) and ventral hippocampus (vHC). Recording locations are highlighted (red ovals) in cresyl-violet stained coronal brain slices. **p < 0.01.

To be able to examine oscillations across a range of behavioral states, we trained mice in an odor-cued working memory task (Figure 1A). Briefly, mice (*n* = 8) were trained to run on a figure-eight maze in which an odor port was placed at one end of the stem arm. Mice were trained to sample the odor by poking and holding their nose in the odor port for at least 1 s. One of two odors (isoamyl acetate or ethyl acetate) was randomly chosen and delivered during the nose poke. Mice then had to retain information about the odor identity while running to the opposite end of the stem arm to make a correct choice – turn left in response to one of the odors or right in response to the other odor. The association between odor and turn direction was held constant for each mouse. Correct choices were rewarded with a single chocolate sprinkle. As expected, initial performance was at chance level (*n* = 8 mice, *Z* = 0.49, *p* = 0.31, one-tailed Wilcoxon signed-rank test). Using a criterion of 65% correct during at least 2 of 3 consecutive days, mice learned the task within 15 ± 5 days. Predictably, performance during the last 3 days of testing was better than during the first 3 days (median: 69.5% vs. 50.2% correct, *n* = 8 mice, *Z* = 2.45, *p* = 0.007, one-tailed Wilcoxon signed-rank test; Figure 1B). All analyses of electrophysiological data were performed on data from the last three testing days for each animal. Earlier recording days were not used because brain activity cannot be considered memory-related while mice do not perform above chance. Recording sites in the OB, mPFC, dHC and vHC were confirmed in histological material (Figure 1C, Supplementary Figure 1). Since histological confirmation of electrode locations was not successful in one animal, we included 7 of 8 animals for all LFP analysis.

### Predominant OB frequencies ranged from 3 to 12 Hz in all task phases

**FIGURE 2:**
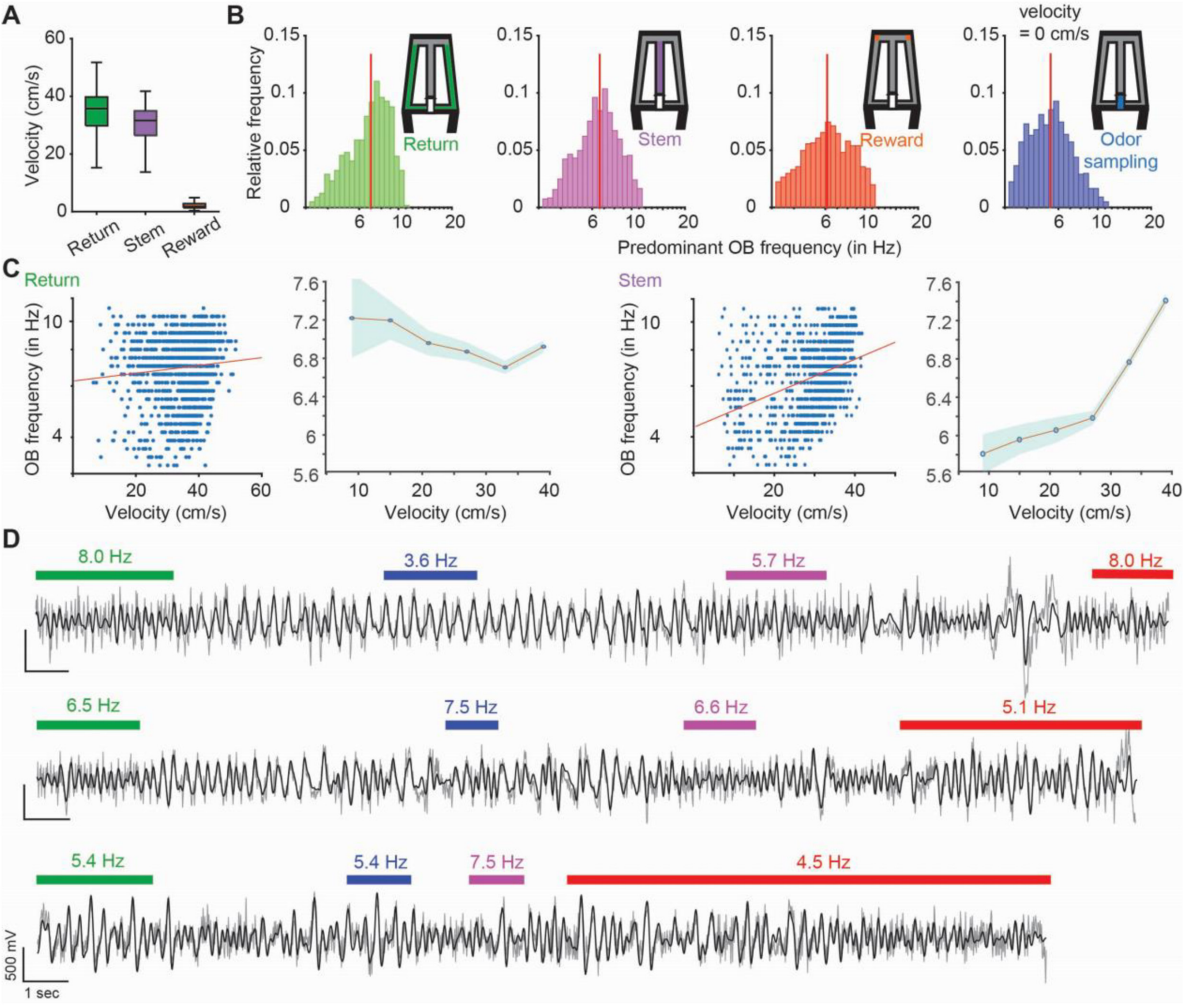
Olfactory bulb (OB) frequencies within each task phase ranged from 3 to 12 Hz. **(A)** Velocity of the mice (*n* = 7 mice, 1207 trials throughout the last 3 days of testing in the odor-guided task) in each maze zone. Velocity in the odor port is not shown and was near zero while animals held their nose in the port during odor sampling. In the box plots, the center line shows the median, and the bottom and top edges of the box represent the 25th and 75th percentiles, respectively. The whiskers indicate the most extreme data points. **(B)** The distribution of predominant OB frequencies across trials is plotted for each task phase. Red vertical line, median. **(C)** In task segments with substantial running, OB oscillation frequency is correlated to different extents with running speed (return: *r* = 0.09, *p* = 0.0017; stem: *r* = 0.33, *p* = 2.78e-31). In the plots depicting averages, dots are means and the shaded area is the standard error of the mean. **(D)** Example OB LFP traces (gray: raw traces, black: 3–12 Hz filtered traces). Each line is a trial, and colored bars indicate time periods when animals were in the respective task phase (green: return arm, blue: odor sampling, purple: stem arm and red: reward zone). Numbers on top of bars indicate the predominant OB frequency. Transition phases are without bars and were not analyzed.

The task was parsed into four phases with distinct behavior patterns – return arms, where animals returned from the chosen reward to the odor port, odor sampling, when animals actively sampled an odor at the odor port, stem arm, where animals ran after odor sampling and while making a choice, and two reward zones, where mice were rewarded if choosing the correct one. Time periods when animals transitioned between these phases were not considered. During the odor sampling period, the animals poked their noses into the odor port and sampled the odor while holding the nose in the odor port and were thus stationary. In the reward zone, the analysis was restricted to periods with low velocity (less than 5 cm/s), so that the mice were mostly, although not completely stationary. Conversely, we confirmed that running speeds on the return arms and on the stem were high and matched (Figure 2A), which allowed us to compare two task phases with corresponding movement patterns.

As expected, the velocity profiles ranged from high running speeds on the stem and return arms to minor movement in the reward zone (Figure 2A), in addition to immobility while in the odor port. For OB oscillations, a frequency range from at least 3 to 12 Hz was observed for each of the behavior phases (Figure 2B) with minor differences in the frequency distributions across the four task phases (*n* = 1207 trials, median ± iqr in return: 7.02 ± 2.77 Hz; stem: 6.58 ± 2.60 Hz; reward zone: 6.16 ± 3.26 Hz; odor sampling: 5.41 ± 2.42 Hz; return vs. odor sampling: *p* = 2.74e-71, KS = 0.37; return vs. stem: *p* = 4.8e-9, KS = 0.13; return vs. reward zone: *p* = 2.57e-15, KS = 0.17; odor sampling vs. stem: *p* = 4.95e-42, KS = 0.28; odor sampling vs. reward zone: *p* = 1.9e-20, KS = 0.20; stem vs. reward zone: *p* = 2.2e-4, KS = 0.09). For task phases with substantial running, some of the variability in OB frequencies–which are corresponding to respiration rate–could be explained by running speed (return: *r* = 0.09, *p* = 0.0017; stem: *r* = 0.33, *p* = 2.78e-31; Figure 2C). Within each trial–starting at the odor port and ending with the mouse returning to the odor port–predominant OB frequencies could vary across task phases (Figure 2D) with significant, but only weak correlations among them (*n* = 1207 trials; return vs. odor sampling: *r* = 0.11, *p* = 9.4e-5; return vs. stem: *r* = 0.49, *p* = 2e-74; return vs. reward zone: *r* = 0.25, *p* = 1.05e-17; odor sampling vs. stem: *r* = 0.17, *p* = 4.2e-9; odor sampling vs. reward zone: *r* = 0.14, *p* = 1.84e-6; stem vs. reward zone: *r* = 0.24, *p* = 1.5e-61, Spearman correlation coefficients; Supplementary Figure 2). The low correlation values among adjacent maze segments exclude the possibility that breathing rates were at a consistent level for sustained periods (e.g., throughout the entire trial or longer), and we therefore used maze segments within trials as the unit for further analyses. OB oscillations in our analyzed frequency ranges have been firmly established as being generated by respiration (Phillips et al., 2012; Rojas-Libano et al., 2014; Jessberger et al., 2016), and we confirmed that this was also the case in our mouse line (Supplementary Figure 3 and Supplementary Table 2). We thus refer to these oscillations as respiration-related oscillations (RROs).

### RROs and canonical theta differed in their frequency ranges

**FIGURE 3:**
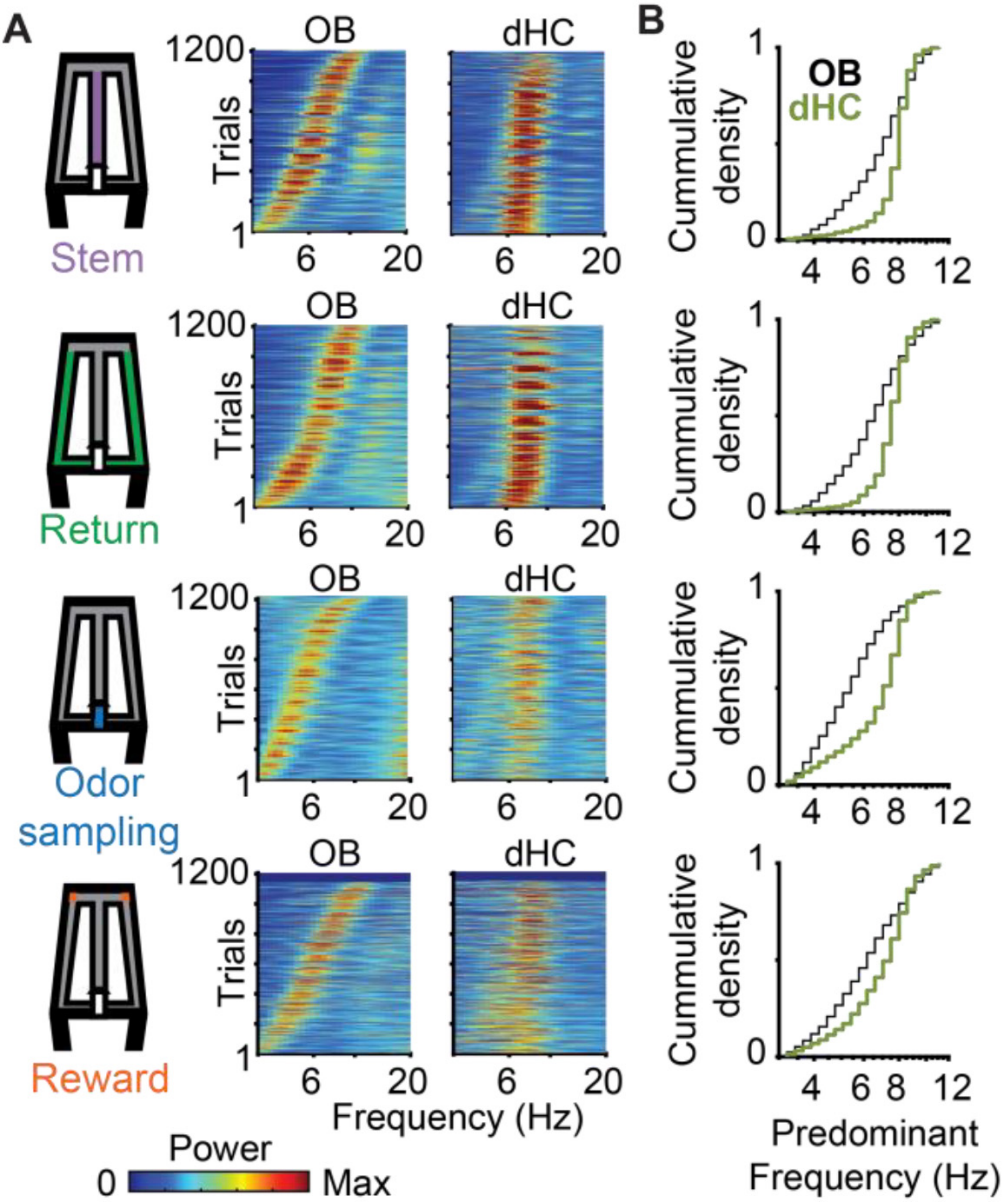
Across trials, predominant OB oscillation frequencies varied so that they were either overlapping or non-overlapping with canonical theta frequencies in dHC. **(A)** Power spectra of OB and dHC oscillations are shown as color-coded plots, with each line corresponding to a trial. Trials are ordered by the OB peak oscillation frequency. **(B)** Cumulative density functions of the predominant OB frequencies (black) and dHC frequencies (green) for the four task phases. The data for OB frequencies is replotted from Figure 2A for comparisons with dHC frequencies. Predominant dHC frequencies were concentrated in the range of 7–11 Hz, while OB frequencies spanned the entire range of 3–12 Hz during all task phases. Frequency distributions differed between brain regions in all task phases (*n* = 1207 trials, return: *p* = 3.4e-56, KS = 0.32; stem: *p* = 1.8e-70, KS = 0.36; odor sampling: *p* = 7.1e-88, KS = 0.41; rewards zone: *p* = 4.7e-21, KS = 0.20).

Because our task design included phases with running and immobility, it allowed us to assess the occurrence of RROs and of canonical theta oscillations in task phases with different movement patterns. As expected for movement-related theta, high amplitude oscillations were observed during periods of running on the stem and return arms (Figure 3A, Supplementary Figure 4A). Hippocampal theta oscillations were also observed during odor sampling, and because mice were stationary while holding the nose in the odor port, theta oscillations during this task phase can be considered sensory evoked (Figure 3A, Supplementary Figure 4B). Sensory-evoked theta oscillations were lower in amplitude than movement-related theta oscillations (Figure 3A; *n* = 7 mice; Friedman Test comparing Return, Stem and Odor sampling: *p* = 6e-7; *Post hoc* Kruskal Wallis: Return vs. Odor sampling *p* = 0.004, Stem vs. Odor sampling *p* = 2.1e-7), but could nonetheless be clearly detected in dHC as distinct from RROs based on their frequency distribution (Figure 3B, Supplementary Figures 4C, D). The predominant frequencies of hippocampal oscillations during odor sampling were >7 Hz, while simultaneously recorded OB oscillations varied more widely in frequency (Figure 3B). Hippocampal theta oscillations were also detected in the reward zone, but this period included immobility and bouts with movement at low velocity (<5 cm/s) such that the type of canonical theta cannot be clearly classified. For all behavior phases, the recorded OB frequencies were thus distributed across the entire 3–12 Hz range while canonical dHC theta was mostly concentrated in the 7–11 Hz range (Figure 3B).

**FIGURE 4:**
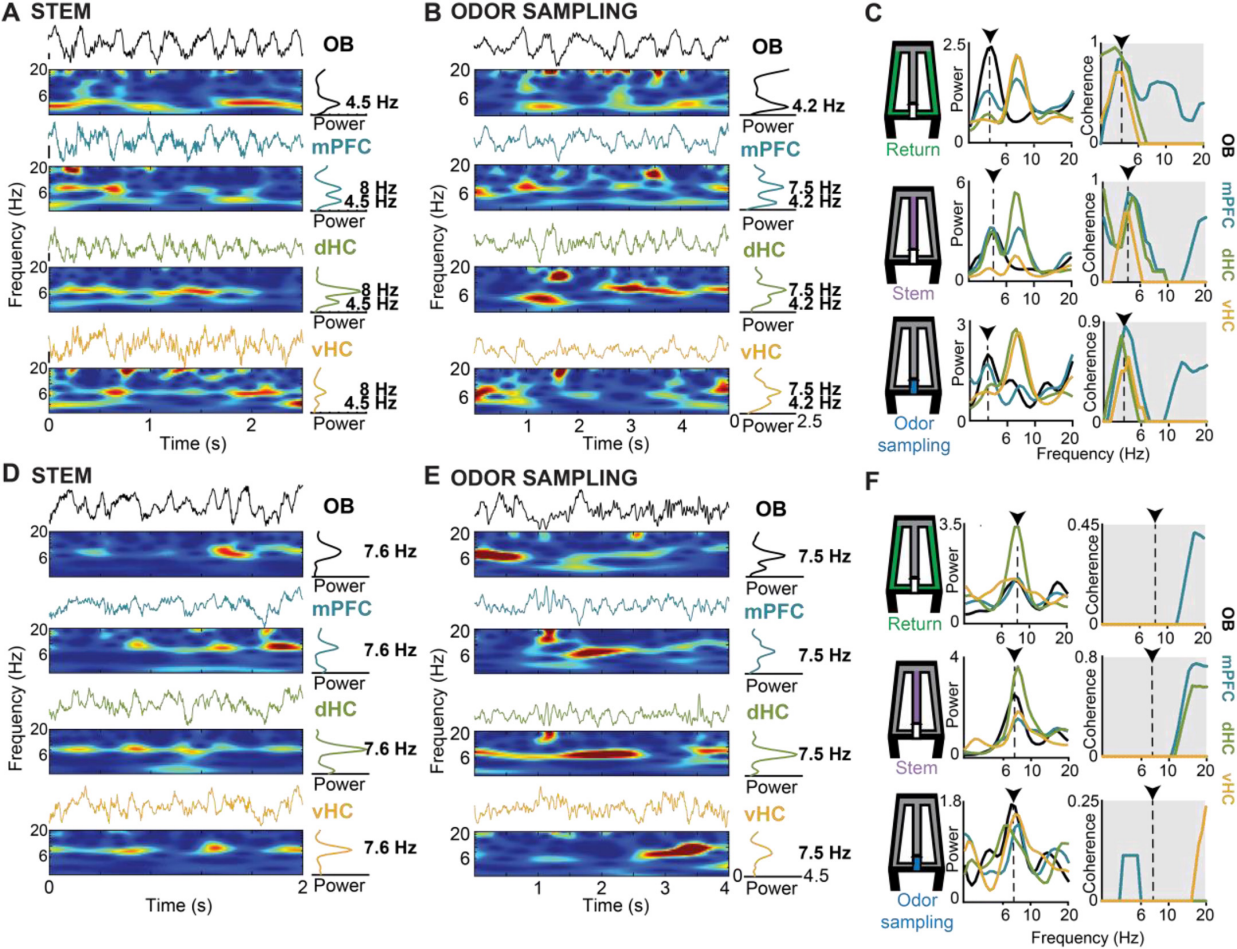
Respiration-related oscillations (RROs) were observed in the mPFC-dHC-vHC network in parallel with movement-related or sensory-evoked theta oscillations. **(A)** Example raw traces and corresponding time-frequency spectrograms of simultaneously recorded LFP from OB, mPFC, dHC and vHC are shown for a period when the animal was running on the stem arm of the maze. In the example, theta frequency in the mPFC-dHC-vHC regions was non-overlapping with the predominant OB frequency. **(B)** Arranged as in panel **(A)** but for an example period when the mouse was stationary while actively sampling odor at the port. As in panel **(A)**, theta and OB frequencies were non-overlapping. **(C)** Time averaged power spectra (left) and coherence spectra (right) are shown for three example periods within a trial and maze zone (return arm, stem arm and odor sampling period) when OB and canonical theta frequencies were non-overlapping. Dotted lines and arrows indicate the frequency of the predominant OB oscillation in the respective trials. OB-mPFC, OB-dHC and OB-vHC coherence is higher at the frequency matching the predominant OB frequency compared to the theta frequency. **(D,E)** Arranged as in panels **(A,B)**, respectively, but for example periods when OB and canonical theta oscillations overlapped in frequency. **(F)** Arranged as in panel **(C)** but for example periods (return arm, stem arm and odor sampling period) with overlapping OB and canonical theta frequencies. Despite the similar peak frequencies of both types of oscillations, OB-mPFC, OB-dHC and OB-vHC coherence was low at the overlapping frequency range. This is consistent with a lack of coupling between canonical theta oscillations and respiration-entrained oscillations in the mPFC-dHC-vHC network.

The wider frequency range for OB compared to canonical theta oscillations implied that there were trials in which the predominant OB frequency and the canonical dHC theta frequency either differed or overlapped. Therefore, we first grouped trials into two categories – trials with overlapping dHC theta and OB frequency (≤1 Hz apart) and trials with non-overlapping dHC theta and OB frequency (>1 Hz apart) (Figure 4). Grouping of trials as overlapping or non-overlapping in frequency was done independently for each task phase within a trial. For example, a trial could be grouped as overlapping for analysis on the stem arm and as non-overlapping for analysis on the return arm. Because behavior in the reward zone includes approach toward the reward, consumption of the reward, and movement initiation after reward consumption, theta could not be firmly classified as either evoked during immobility or movement-related, the analysis focused on the remaining three maze regions – the return arms, odor sampling and stem arm. During each of these three phases, mice were either running (i.e., return arms, stem arm) or stationary (i.e., odor sampling), and canonical theta oscillations (>7 Hz) were thus considered to be either movement-related or sensory-evoked (Figure 3). With canonical theta oscillations >7 Hz, most trials with OB frequencies <7 Hz were non-overlapping (93.3%, 91.9%, and 85.7 % in return, odor sampling and stem; Supplementary Table 1). However, for OB frequencies above 7 Hz, trials in which OB frequency and hippocampal theta frequency either overlapped (frequency difference < 1 Hz) or did not overlap were more evenly distributed (non-overlapping: 34.3%, 37.6% and 27.8% in return, odor sampling and stem; Supplementary Table 1). The data were therefore analyzed for the three combinations with a substantial number of trials (non-overlapping < 7 Hz, non-overlapping ≥ 7 Hz, overlapping ≥ 7 Hz; *n* = 475, 224, 430 in return, *n* = 780, 88, 146 in odor sampling, *n* = 526, 141, 367 in stem).

### Coherence with OB oscillations occurred irrespective of task phase

**FIGURE 5:**
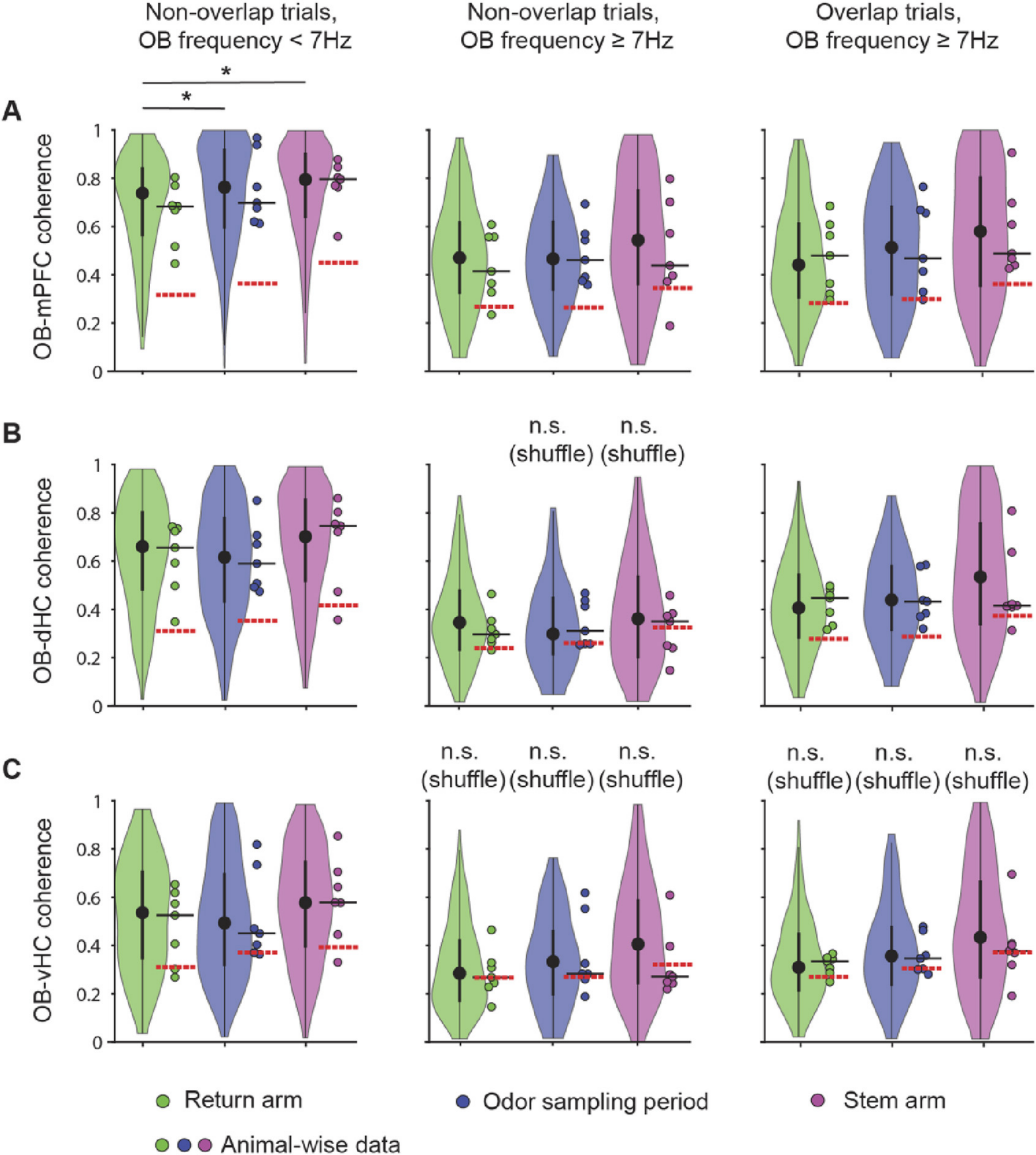
Coupling of cortical areas with OB oscillations occurred in all task phases and at high and low RRO frequencies. Violin plots of OB-mPFC **(A)**, OB-dHC **(B)**, and OB-vHC **(C)** coherence in the return arm, the odor sampling period, and the stem arm. Non-overlap trials with RRO frequency < 7 Hz, non-overlap trials with RRO frequency ≥ 7 Hz and overlap trials with RRO frequency ≥ 7 Hz were analyzed separately. Coherence in each trial was calculated at the RRO frequency. Significant differences between task phases were found only for OB-mPFC coherence with OB frequency < 7 Hz [*n* = 7 mice, OB-mPFC: $\chi_{F}^{2}$ (1) = 7.18, *p* = 0.028; OB-dHC: $\chi_{F}^{2}$ (1) = 2.33, *p* = 0.31; OB-vHC: $\chi_{F}^{2}$ (1) = 2.58, *p* = 0.27, Friedman Test]. *Post hoc* Wilcoxon Rank Sum tests with a Bonferroni-Holm correction was performed to determine that coherence was higher in odor sampling and stem compared to return. In addition, coherence was higher with RROs at low (<7 Hz) compared to high frequencies (≥7 Hz), for mPFC and vHC irrespective of overlap, but for dHC, depending on overlap (see text and Supplementary Table 3 for details). Violin plots: center circle, median; bottom and top of thick vertical line, 25th and 75th percentile, horizontal width, relative frequency. Dots with a black outline, data from individual mice; thick black horizontal line; median of animal-wise data; red dashed horizontal line, chance level of coherence obtained from shuffling pairs of LFP signals across trials. *p < 0.05.

We first analyzed the relation between RROs and theta oscillations across task phases. With OB oscillations < 7 Hz, LFP signals at prefrontal and hippocampal recording sites typically showed two detectable peaks during running on the stem and in return arms – one at a frequency of ∼8 Hz (i.e., canonical theta) and another matching the predominant OB frequency (Figures 4A, C). Similarly, LFP in the prefrontal-hippocampal network showed two peaks while mice were stationary during odor sampling periods (Figures 4B, C). Given that peaks at the respiration frequency – albeit smaller in amplitude than in OB – could be detected in mPFC, dHC, and vHC, we asked whether the oscillations at each of the cortical recoding sites were coupled to OB oscillations. Because there were differences in OB amplitude between trials in which RRO frequency and canonical theta frequency were either overlapping or not (Supplementary Figure 5 and Supplementary Table 3), we used a coherence measurement that is independent of amplitude (see Section “Materials and Methods”). The maxima of the coherence spectra were often observed at frequencies that at least approximately matched the peak OB frequency (Figure 4C), and coupling of cortical regions at OB frequencies was generally detectable in all task phases and irrespective of OB frequency (<7 Hz or ≥7 Hz) or overlap of OB frequency with canonical theta frequency (Figures 4D–F, 5, Supplementary Figure 6). There were only few exceptions when coherence did not exceed chance levels (e.g., OB-vHC at OB frequencies ≥ 7 Hz; Figure 5). RROs can thus be coherent between OB and mPFC and between OB and hippocampus in all task phases and at low and high respiration frequencies. We tested for sex differences in this effect and did not find any (Supplementary Figure 7). Data were therefore pooled for this and other analyses.

**FIGURE 6:**
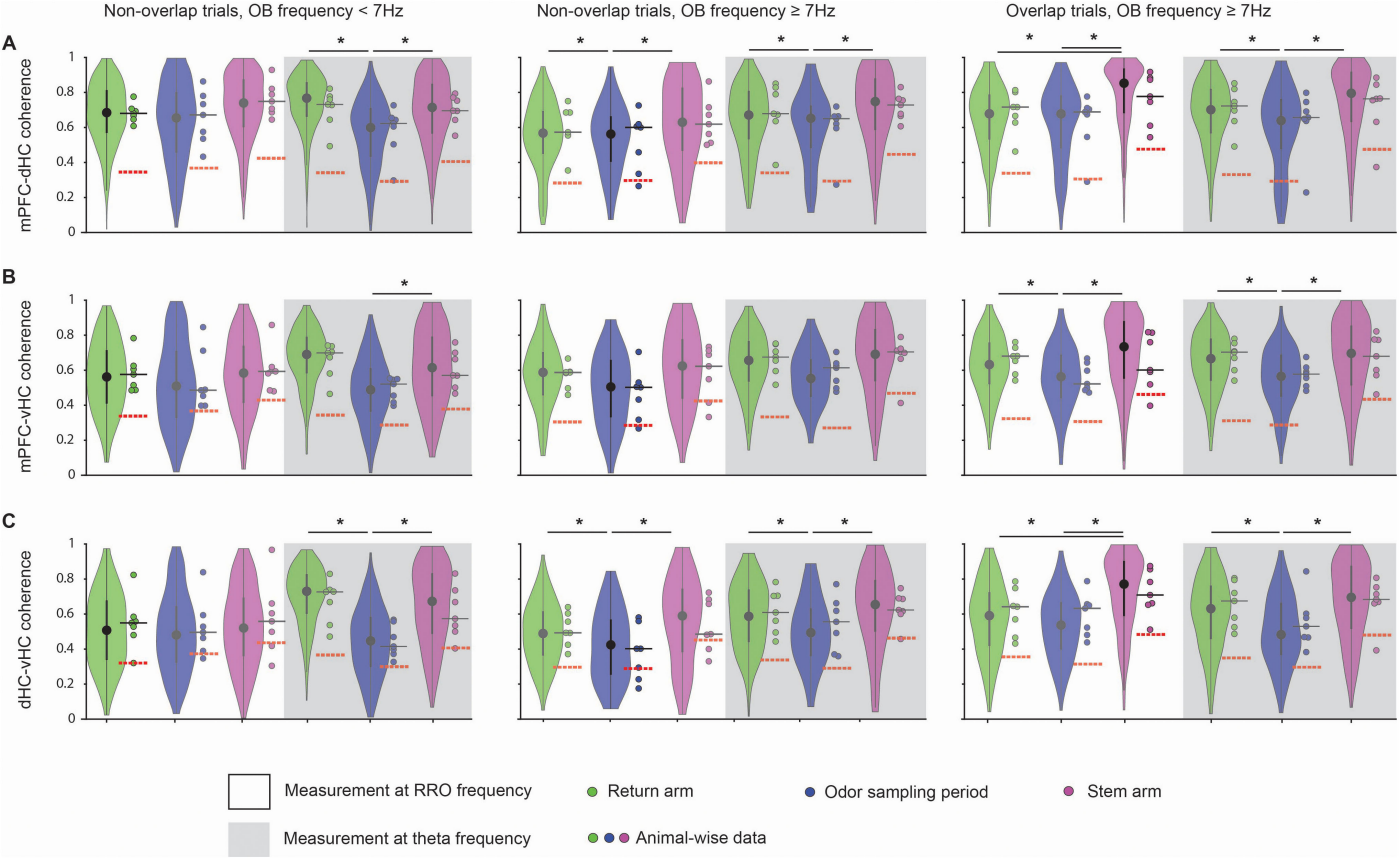
Coherence between cortical regions at the RRO frequency was high at low and high RRO frequencies. Violin plots of the coherence at peak RRO frequency (measured in OB) and at the peak canonical theta frequency (measured in hippocampus) for mPFC-dHC pairs **(A)**, mPFC-vHC pairs **(B)**, and dHC-vHC pairs **(C)**. Coherence across all three pairs of regions was compared between the return arms, odor sampling and the stem arm. Intrahippocampal coherence of canonical theta oscillations was often higher in the stem arm and the return arms than during odor sampling (see Supplementary Table 4 for detailed statistics). Violin plots: center circle, median; bottom and top of thick vertical line, 25th and 75th percentile, horizontal width, relative frequency. Dots with a black outline, data from individual mice; thick black horizontal line; median of animal-wise data; red dashed horizontal line, chance level of coherence obtained from shuffle analysis. *p < 0.05.

The finding that coupling of multiple brain regions to OB at RRO frequencies is strong and widely observed across task phases raises the question whether there is also coupling between cortical regions at RRO frequencies in different phases of the olfactory working memory task. For comparison, coupling at the canonical theta frequency was measured. The question of the relative importance of coupling at one or the other frequency type is of particular interest for mPFC where both frequencies are prominent. In addition, it is also feasible that coupling increased when the two oscillations overlapped in frequency and might be entrained to each other. To consider these possibilities, we compared coherence measurements at the RRO/theta frequency during trials with overlapping and non-overlapping RRO and theta frequencies. Interestingly, we observed that coupling at RRO frequency as well as at canonical theta frequency was high and significantly above chance between mPFC and dHC as well as mPFC and vHC during all task phases. The coupling at RRO frequency occurred irrespective of whether OB frequency was high or low, and when OB frequency was above 7 Hz, irrespective of whether the OB frequency overlapped with canonical theta frequency or not (Figures 6A–C and Supplementary Table 4). Coupling at RRO frequency was even observed between dHC and vHC (Figure 6C). Similarly, coherence at the canonical theta frequency was high across cortical regions in all task phases. A notable exception is the lower coherence during odor sampling than in other task phases, in particular for mPFC-dHC and for dHC-vHC pairs (Figure 6). Because this effect was observed for the canonical theta frequency, it is unsurprising that the reduced coherence during odor sampling was observed irrespective of OB frequency (<7 Hz or ≥7 Hz; Figure 6).

Our results are not consistent with the possibility that coherence is generally higher when RRO power is higher at lower frequencies (Supplementary Figure 3B) because coherence could be highest at RRO frequencies ≥ 7 Hz (e.g., coherence between mPFC and dHC in the stem; Figure 6A). Furthermore, trials when OB oscillations were ≥7 Hz allowed for a comparison of coherence between trials with overlapping and non-overlapping RRO/theta oscillation frequencies without having to include measurements at lower frequencies. For all task phases and pairs of brain regions in trials with RRO frequency ≥ 7 Hz, coherence was of comparable amplitude irrespective of whether the two types of oscillations occurred at overlapping or non-overlapping frequencies (Figures 6A–C).

Although the detection of differences in coherence patterns across task phases and trial types already suggest that coherence between OB and each of the cortical regions is not simply a consequence of volume conduction or LFP power, we also used other measurements to exclude this possibility. First, we measured phase differences across recording sites. For all pairs of brain regions, the phase difference was significantly different from zero in few if not most combinations of task phases and trial types (Supplementary Figure 8). Furthermore, our coherence measures did not change in parallel with oscillation amplitude, as we often observed no differences between coherence in trials with overlapping compared to non-overlapping frequencies despite a change in power at participating recording sites (Figure 6, Supplementary Figure 5). Also, a comparison of running velocity in trials with overlapping and non-overlapping frequencies did not reveal any differences [return: *n* = 7 mice, $\chi_{F}^{2}$ (1) = 2.45, *p* = 0.30, stem: *n* = 7 mice, $\chi_{F}^{2}$ (1) = 3.88, *p* = 0.14, Friedman test], such that velocity-related amplitude differences of oscillations could not have contributed to balancing effects. Taken together, these results and our use of a coherence measurement that is independent of power suggest that coherence across brain regions was not trivially related to either volume conduction or oscillation amplitude.

### Coherence between prefrontal cortex and hippocampal regions was unrelated to odor-guided memory performance

Even though we examined odor-cued working memory in our task, the figure-eight maze is often used to assess spatial alternation behavior in rodents. While we did not train the mice to alternate in the odor-cued task, we observed spatial alternation on successive trials (i.e., right turn followed by a left turn or vice versa) in the beginning of behavioral training when the odor-cued choice behavior was at chance (data not shown). We reasoned that mice’s propensity toward spatial alternation may continue to interfere with odor-cued choices even after the mice performed above chance in the odor-cued version. To test this possibility, we analyzed – throughout the last 3 testing days – four different combinations of trial types – alternating correct, alternating incorrect, non-alternating correct, non-alternating incorrect – with correct and incorrect referring to the odor-guided response and alternating and non-alternating referring to the turn direction compared to the previous choice. Although not rewarded, alternation behavior was above chance (*n* = 8 mice, *Z* = 2.28, *p* = 0.011, one-tailed Wilcoxon signed-rank test) on the same testing days when odor-guided choices were also above chance (see Figure 1). However, there was no interaction with the odor-guided responses [correct odor-guided responses in 68.8% of trials; alternation behavior in 63.8% of trials; χ^2^ (1, 1207) = 0.01, *p* = 0.92].

**FIGURE 7:**
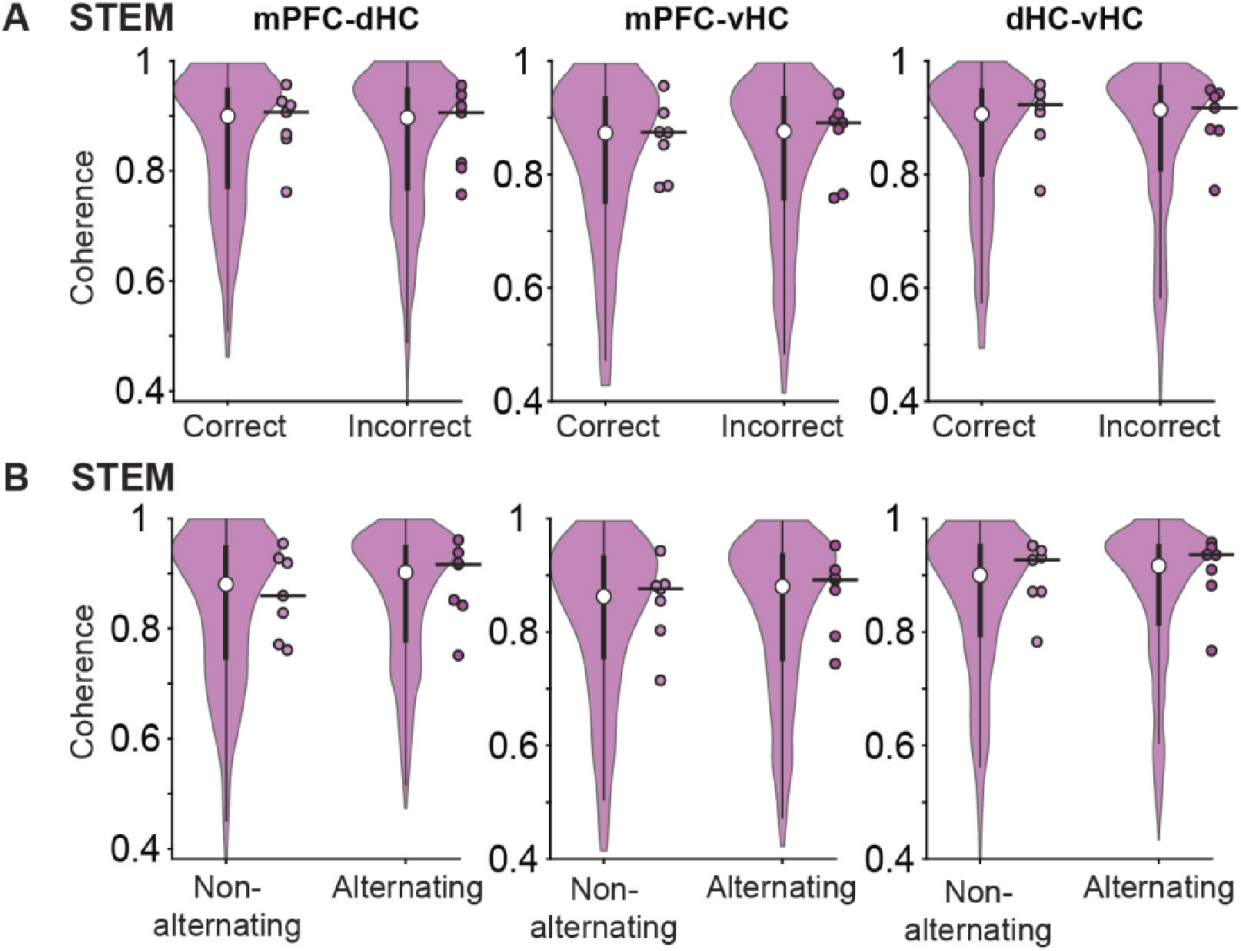
Movement-related theta oscillations in the stem arm were highly coherent across cortical regions during correct and incorrect choices and during alternating and non-alternating choices. **(A)** Coherence of movement-related theta oscillations between pairs of regions in the mPFC-dHC-vHC network in the stem arm is compared between trials with correct and incorrect choices. Coherence was not different between trials with correct and incorrect choices (see text for statistics). **(B)** Same as panel **(A)**, but for alternating compared to non-alternating choices. Coherence was not different between trials with alternating choices compared to trials with non-alternating choices (see text for statistics). Violin plots: center circle, median; bottom and top of thick vertical line, 25th and 75th percentile, horizontal width, relative frequency. Dots with a black outline, average data from individual mice; thick black horizontal line; median of animal-wise data.

Given that we observed choices that were guided by the odor and also choices that were consistent with alternation above chance, we analyzed the LFP signal that occurred immediately preceding the choice point (i.e., on the stem arm) across different types of trials. Coherence between pairs of regions in the mPFC-dHC-vHC network in the stem arm was not different between trials with correct and incorrect odor-cued responses [*n* = 7 mice, mPFC-dHC: $\chi_{F}^{2}$ (1) = 0.01, *p* = 0.93; mPFC-vHC: $\chi_{F}^{2}$ (1) = 0.01, *p* = 0.93; dHC-vHC: $\chi_{F}^{2}$ (1) = 0.01, *p* = 0.93, Friedman test; Figure 7A]. While mPFC-dHC coherence was slightly higher during trials with alternating choices compared to same-side (i.e., non-alternating) choices, repeated-measures statistics did not reveal any significant effects [*n* = 7 mice, mPFC-dHC: $\chi_{F}^{2}$ (1) = 0.17, *p* = 0.68; mPFC-vHC: $\chi_{F}^{2}$ (1) = 1.97, *p* = 0.16; dHC-vHC: $\chi_{F}^{2}$ (1) = 0.82, *p* = 0.36, Friedman test; Figure 7B]. Coherence of canonical theta in the prefrontal-hippocampal network is therefore unrelated to odor-guided or spatially guided behavior when spatially guided behavior is not reinforced.

**FIGURE 8:**
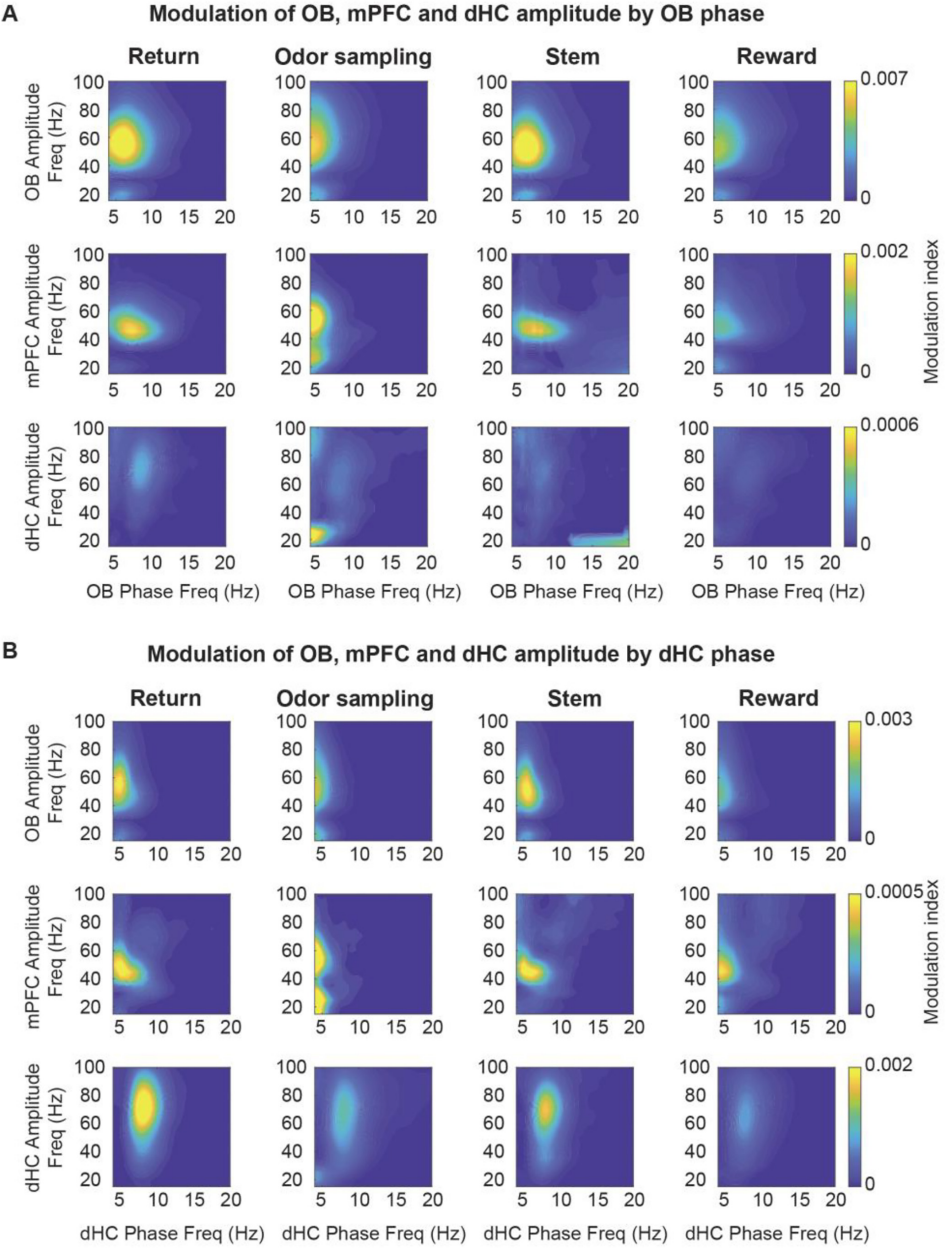
The amplitude of gamma and beta oscillations in OB, mPFC and dHC was modulated by the phase of OB and theta oscillations. Coupling of OB, mPFC and dHC oscillation amplitude (20–100 Hz) to the phase of OB oscillations **(A)** and theta oscillations **(B)**. Beta oscillations in the OB were modulated by OB phase in all task phases. Beta oscillations in the mPFC and dHC were modulated by the phase of OB and canonical theta oscillations, but predominantly during odor sampling. Low gamma (30–60 Hz) amplitude in OB and mPFC was modulated by OB and canonical theta phase in all task phases. Mid gamma (60–100 Hz) amplitude in dHC was predominantly modulated by canonical theta phase, and this modulation was more pronounced during movement on the return and stem arms. Color scale, modulation index.

Because coupling at RRO and theta frequencies was detected in all behavior phases, we next examined whether each of these oscillations may show selective cross-frequency coupling across brain regions in any of the task phases. By calculating phase-amplitude coupling, we first confirmed the strong coupling of local low gamma (∼40–60 Hz) and beta (∼20 Hz) in OB to OB oscillation phase, as previously reported (Kay and Freeman, 1998; Kay et al., 2009; Kay and Lazzara, 2010; Tort et al., 2025). The coupling was observed in all behavior phases (i.e., return arms, odor port, stem, rewards zone). A similar pattern of coupling to OB oscillations as for local gamma and beta within OB was observed for gamma and beta in mPFC, which indicates that mPFC fast oscillations primarily couple to the phase of RROs (Figure 8A). The likely coupling of mPFC oscillations to respiratory-related oscillations was also inferred by Tavares and Tort (2022) and is shown here with direct recordings of OB oscillations. In contrast, coupling of gamma and beta oscillations to OB oscillation phase showed a different pattern in the hippocampus. Whereas coupling of gamma and beta to RRO phase occurred in OB and PFC in all behavioral phases, it only occurred during odor sampling and only for beta oscillations in hippocampus (Figure 8A). For comparison, we also analyzed cross-frequency coupling to the hippocampal canonical theta oscillations. For canonical theta, we observed differences between periods with movement theta and sensory-evoked theta. Movement theta showed higher theta-gamma coupling than sensory-evoked theta. However, coupling to beta phase was, as for RROs, strongest during odor sampling (Figure 8B). Given that the modulation index for beta oscillation during odor sampling is several-fold lower when using hippocampal rather than OB oscillations as a reference signal while beta oscillations are, even with the hippocampal reference signal, modulated by ∼4 Hz oscillations, beta amplitude is likely modulated by hippocampal RROs, although these are much lower in amplitude than the more prominent canonical theta oscillations.

## Discussion

Synchronized oscillations are thought to facilitate coordinated computations across brain regions. Although it is well established that respiration-entrained oscillations are propagated from the OB to other cortical areas, it is unclear to what extent the respiration-entrained oscillations interfere with or are synergistic with canonical theta oscillations in the prefrontal-hippocampal regions. If coupling occurs, it would be an indication that oscillations that are generated by two different mechanisms in the brain could dynamically couple to support memory computations. To investigate the coordination of respiration-entrained oscillations in the OB and of theta oscillations in the prefrontal-hippocampal circuit, we analyzed simultaneously recorded LFP signals from the OB, mPFC, dHC and vHC during an odor-cued working memory task. We found that respiration-entrained oscillations in the OB were distributed across the 3–12 Hz frequency range within each task phase, including phases when animals moved and phases when animals were predominantly immobile. By examining these task phases separately, we were able to test whether movement-related and sensory-evoked canonical theta oscillations (∼8 Hz) in the prefrontal-hippocampal circuit interact with respiration-entrained oscillations. We found that coherences – of OB with cortical regions and among cortical regions – at both the RRO frequency and at the canonical theta frequency was high during all task phases. The coherence values were generally similar irrespective of whether RROs occurred at a different frequency than canonical theta or at a frequency overlapping with theta. Taken together, respiration-entrained oscillations were thus propagated from the OB to prefrontal-hippocampal regions and couple the same brain regions that are also coupled by the canonical theta frequency. During odor sampling, when olfactory inputs can be assumed to strongly drive information processing, coherence at RRO frequencies generally remained as high as in other task phases, while intrahippocampal coherence at the canonical theta frequency decreased. Therefore, RROs became the frequency with the most prominent coupling during odor sampling.

### Breathing frequency is variable but only weakly controlled by ongoing behavior

It has long been known that rodent breathing frequencies can vary over a wide range. Mice have a “passive” breathing frequency of 1–4 Hz during quiescence (Wesson et al., 2011; Jessberger et al., 2016). Upon exposure to a novel odor, mice begin “active” sniffing at a high frequency of 4–12 Hz (Wesson et al., 2008, 2011; Jessberger et al., 2016). Such a modulation of respiration frequencies during odor sampling has been thought to be the basis for odor processing in lower-order olfactory circuits (Wesson et al., 2008). Indeed, sniffing frequency changes the number of odor molecules arriving at the olfactory sensory neurons, thereby increasing their responsiveness to odors at these higher sniffing frequencies (Courtiol et al., 2011). However, further investigations of the role of sniffing frequencies in odor information processing has revealed that mice have varied strategies in terms of sniffing frequencies (Wesson et al., 2008; Reisert et al., 2020). While sniffing frequencies increase in response to a novel odor sampling, mice are able to perform “easy” as well as “difficult” odor discrimination tasks without a significant increase in their sniffing frequencies compared to baseline (Wesson et al., 2008, 2009). Our results are consistent with a weak control of breathing frequencies by ongoing behavior because we find that a similarly broad range of OB oscillations can occur in any of the behavioral phases in an odor-cued working memory task. Interestingly, we find that coupling of RROs between OB and other brain regions is high at low RRO frequencies, which implies that an upshift into the sniffing frequency range is not required for coordination of oscillations between OB and other brain regions. However, during odor sampling in particular, there is also cross-frequency coupling between RROs and beta frequencies, and this type of coupling seems to be a selective conduit for not only engaging local circuits in OB, but for also coupling to mPFC and the hippocampus at the transition from odor sampling to decision making (Symanski et al., 2022).

### Coupling of movement-related theta and sensory-evoked theta to RROs

Although oscillations in the 4–12 Hz band are broadly referred to as theta, it is well established that theta oscillations in the hippocampus are of at least two types – type I and type II. Type I theta is atropine-insensitive and is movement-related (Vanderwolf, 1969; Kramis et al., 1975). Power and frequency of type I theta oscillations have been shown to increase with higher running speeds (Feder and Ranck, 1973; Kuo et al., 2011). Our analysis of movement-related theta oscillations in the return and stem arms revealed that theta oscillations during those periods showed similar relations to movement as type I theta (Supplementary Figure 4A). On the other hand, type II theta is atropine sensitive and is unrelated to movement (Vanderwolf, 1969; Kramis et al., 1975). Type II theta is elicited when the animal is exposed to arousing, vigilant and aversive conditions, such as a predator’s smell (Sainsbury et al., 1987). Our recording of sensory-evoked theta oscillations during the odor sampling period is akin to type II theta oscillations, although we did not test the atropine sensitivity of these oscillations. However, we confirmed that these theta oscillations occur while the mouse’s nose was held stationary in the odor port (Figures 3A, B, Supplementary Figure 4B), which suggests that theta oscillations during this task phase fulfill at least one of the criteria for type II theta. By including task phases in the analysis when theta was either movement-related or sensory-evoked, we were able to test to what extent each type of theta was related to RROs. We found that movement-related and sensory-evoked theta were both distinct from RROs, which were often below the frequency of either type of theta oscillations. Notably, the coherence across brain regions at the theta frequency was lower for sensory-evoked theta during odor sampling than for movement theta in other task phases. Conversely, coherence at the RRO frequency was high in all task phases (Figure 6). The high coherence at RRO rather than theta frequency during odor sampling is consistent with findings in rats of coordination of brain activity at the respiration rhythm in odor-cued working memory tasks (Fujisawa and Buzsaki, 2011; Symanski et al., 2022).

For movement-related theta oscillations in the hippocampus, seminal work that recorded nasal air flow as well as LFP from the OB and dorsal hippocampus found that the hippocampal movement-related theta does not couple with the respiration rhythm during exploration (Vanderwolf and Szechtman, 1987). Similarly, RROs were identified as a separate oscillation from theta oscillation during running based on differences in the depth profiles across hippocampal recording sites between both types of oscillations (Nguyen Chi et al., 2016). For sensory-evoked theta, there is evidence for coupling to RROs, but it is not equivocal. For example, Macrides et al. (1982) reported that during odor sampling, theta oscillations in the hippocampus couple with the respiration rhythm during the initial stages of learning an odor discrimination reversal task, but that coherence between these oscillations was low in expert animals. Conversely, Kay (2005) showed that hippocampal theta oscillations and the sniffing rhythm were coherent during odor sniffing in a two-odor discrimination task and that the coherence was positively correlated to performance, which suggests that coherence remained high even when animals were proficient. These discrepancies could, at least in part, be explained by the consideration that both canonical theta and RROs can be recorded with hippocampal electrodes. What is interpreted as coherence between hippocampal theta and respiration-entrained OB oscillations could therefore be coherence between OB oscillations and RROs that can be recorded in the hippocampus. Hippocampal RROs are readily detectable (Tort et al., 2018; Figure 4) and are particularly pronounced for electrodes in the dentate gyrus (Yanovsky et al., 2014; Nguyen Chi et al., 2016) and at ventral hippocampal sites. Accordingly, when we focus on the RRO component of our LFP signals, we indeed find that it is strongly coupled to OB oscillations during odor sampling (see Figure 4C), similar to what has been reported during immobility (Nguyen Chi et al., 2016; Tort et al., 2018) and in odor-guided working memory (Symanski et al., 2022). Oscillations that arise from separate pacemakers – nasal air flow for RROs (Onoda and Mori, 1980; Phillips et al., 2012; Wu et al., 2017) and medial septal area for canonical theta (Gaztelu and Buno, 1982; Mitchell et al., 1982; Bland and Bland, 1986) – can thus in parallel result in coupling across large brain systems (Yanovsky et al., 2014; Lockmann et al., 2016; Nguyen Chi et al., 2016; Biskamp et al., 2017; Koszeghy et al., 2018; Jung et al., 2022; Symanski et al., 2022; Tort et al., 2025).

Our results are consistent with existing narratives that respiration-entrained oscillations are detected in the prefrontal-hippocampal areas and can be particularly evident when the respiration frequency is lower than the theta oscillation frequency in the hippocampus (Nguyen Chi et al., 2016). In fact, a majority of investigations have studied periods of low respiration frequency (<6 Hz) (Yanovsky et al., 2014; Lockmann et al., 2016; Nguyen Chi et al., 2016) while respiration frequencies can extend to 10 Hz and above and thus be higher than the canonical theta frequency. However, while mitral and tufted cells in the OB are entrained to the respiration rhythm at low frequencies (up to 6 Hz), they fire tonically at higher respiratory frequencies (6–12 Hz) (Kay and Laurent, 1999). It may therefore be the case that respiration-entrained oscillations in the OB are transmitted differently to downstream cortical areas based on frequency. In the subset of trials with non-overlapping RRO and canonical theta frequencies and when RRO frequency was higher than 7 Hz, we nonetheless found respiration-entrained OB oscillations coupled to hippocampal oscillations well above chance levels. Our results of substantial OB-mPFC coherence and OB-HC coherence at higher frequencies (Figures 5, 6) thus differ from reports that higher breathing frequencies do not as effectively entrain OB cells and as effectively coordinate cortical networks (Kay and Laurent, 1999; Juventin et al., 2023).

### Are coupled oscillators across brain regions related to behavioral performance?

Previous studies firmly established that RROs propagate from the OB to downstream brain regions in a variety of brain states including anesthesia, mobility and immobility (Fontanini et al., 2003; Ito et al., 2014; Yanovsky et al., 2014; Lockmann et al., 2016; Nguyen Chi et al., 2016; Biskamp et al., 2017). Based on these observations, it was speculated that respiration-entrained oscillations are a global signal that synchronizes activity across multiple brain regions and supports sensorimotor integration in a context dependent manner (Macrides et al., 1982; Yanovsky et al., 2014; Lockmann et al., 2016; Nguyen Chi et al., 2016; Tort et al., 2018). However, at least one previous study that tested functional coupling at the theta frequency did not find coherent oscillations between the OB and hippocampus. Because these results were obtained in a simple hippocampus-independent odor discrimination task (Fortin et al., 2002), we considered the possibility that coupling between OB and hippocampal oscillations could emerge in a task that involves the learning of associations between odors and spatial locations, which has been shown to be hippocampus-dependent (Gilbert and Kesner, 2004). Although we did not find that RROs were synergistically coupled to canonical theta oscillations in any behavioral phase, we observed that coupling to RROs becomes more prominent than coupling to canonical theta during odor sampling–when sensory information to support working memory likely engage hippocampal computations. During odor sampling–when coupling at RRO frequency was prevalent–we also found that RRO phase modulated beta oscillations in dorsal hippocampus, as also seen in odor-guided working memory tasks in rats (Fujisawa and Buzsaki, 2011; Symanski et al., 2022). Because beta oscillations are prominent in the lateral entorhinal cortex and because their coherence is related to memory processing (Igarashi et al., 2014), coordination in this frequency range points to a possible pathway from OB to hippocampus through lateral entorhinal cortex. Therefore, our results support a key role of RROs in the coordination of computations for memory-based decision making.

## Data Availability

The raw data supporting the conclusions of this article will be made available by the authors, without undue reservation.
