# Supplementary material for: Oscillations in the prefrontal-hippocampal circuit couple to respiration-related oscillations during all phases of a working memory task
Source: Front Behav Neurosci. 2025 Oct 30;19:1669111. doi: 10.3389/fnbeh.2025.1669111 (PMC12611856; doi:10.3389/fnbeh.2025.1669111)
Supplement: Supplementary file 1 [file Supplementary_file_1.pdf]

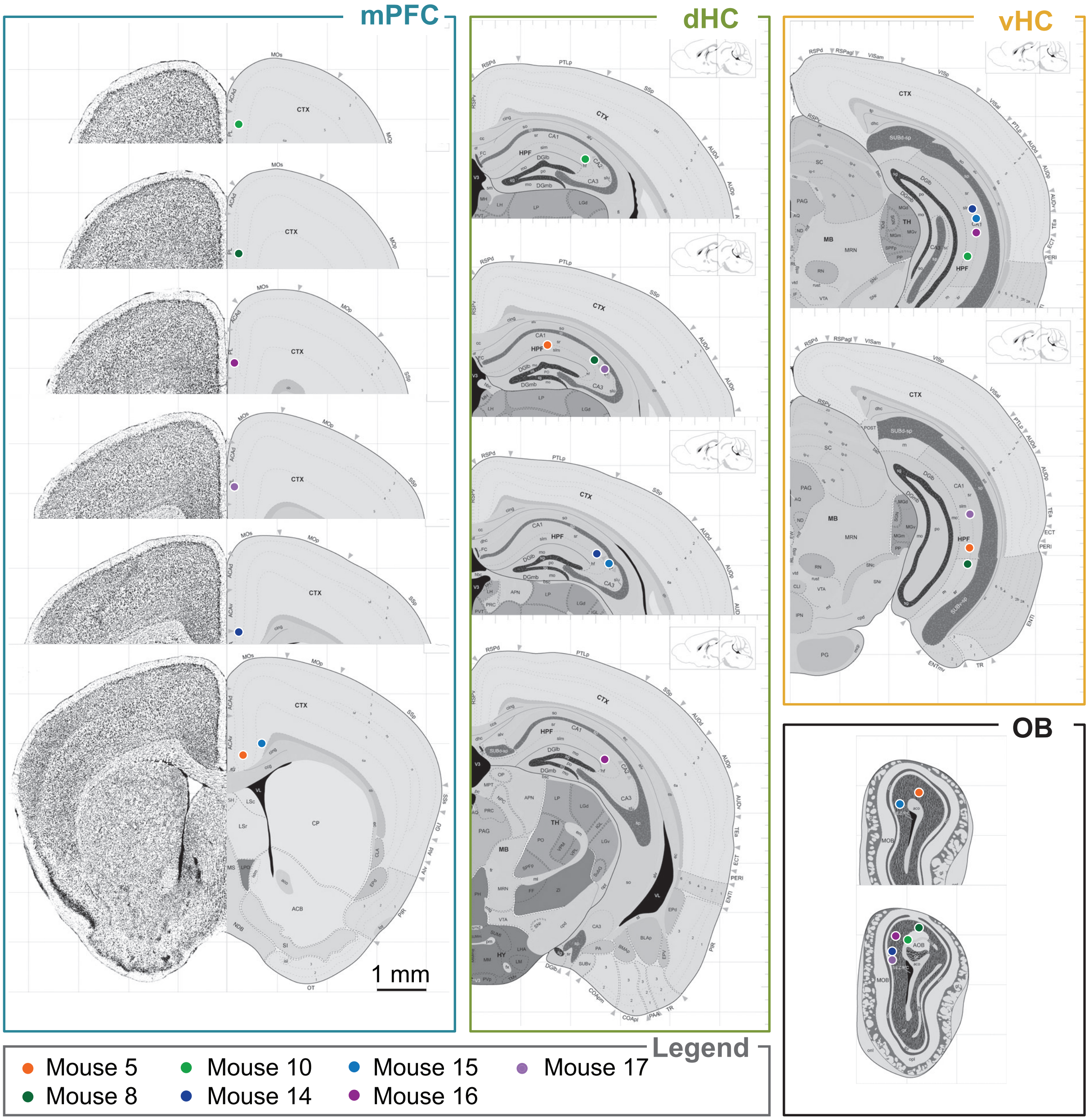

Figure S1. Electrode recording locations. Colored dots indicate the recording locations in each animal (n = 7) used in the study. Electrodes were histologically confirmed to be placed in the OB, the prelimbic, infralimbic and anterior cingulate areas of the mPFC and the CA1 areas of the dHC and the vHC and are shown on coronal sections from the Allen Mouse Brain Atlas.

Allen Institute for Brain Science (2004). Allen Mouse Brain Atlas [dataset]. Available from mouse.brain-map.org. Allen Institute for Brain Science (2008).

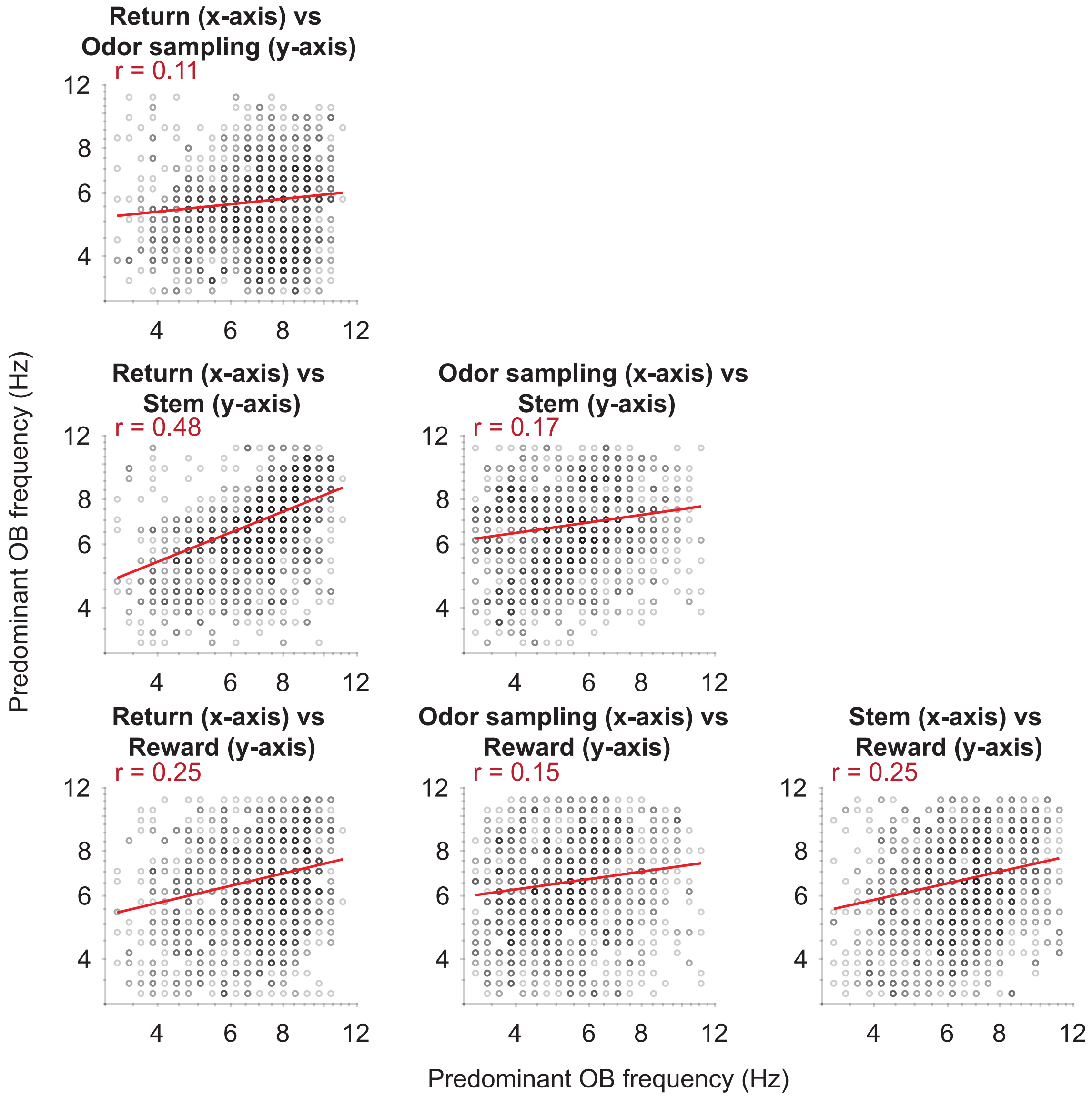

Figure S2. Weak correlations between predominant OB frequencies in different task phases. Scatter plots of predominant OB frequencies during different task phases in each trial. Spearman correlations between predominant OB frequencies during different task phases were weak. Spearman correlation coefficients: Return vs Odor sampling: r = 0.11, p = 9.4e-5; Return vs Stem: r = 0.49, p = 2e-74; Return vs Reward: r = 0.25, p = 1.05e-17; Odor sampling vs Stem: r = 0.17, p = 4.2e-9; Odor sampling vs Reward: r = 0.14, p = 1.84e-6; Stem vs Reward: r = 0.24, p = 1.5e-61.

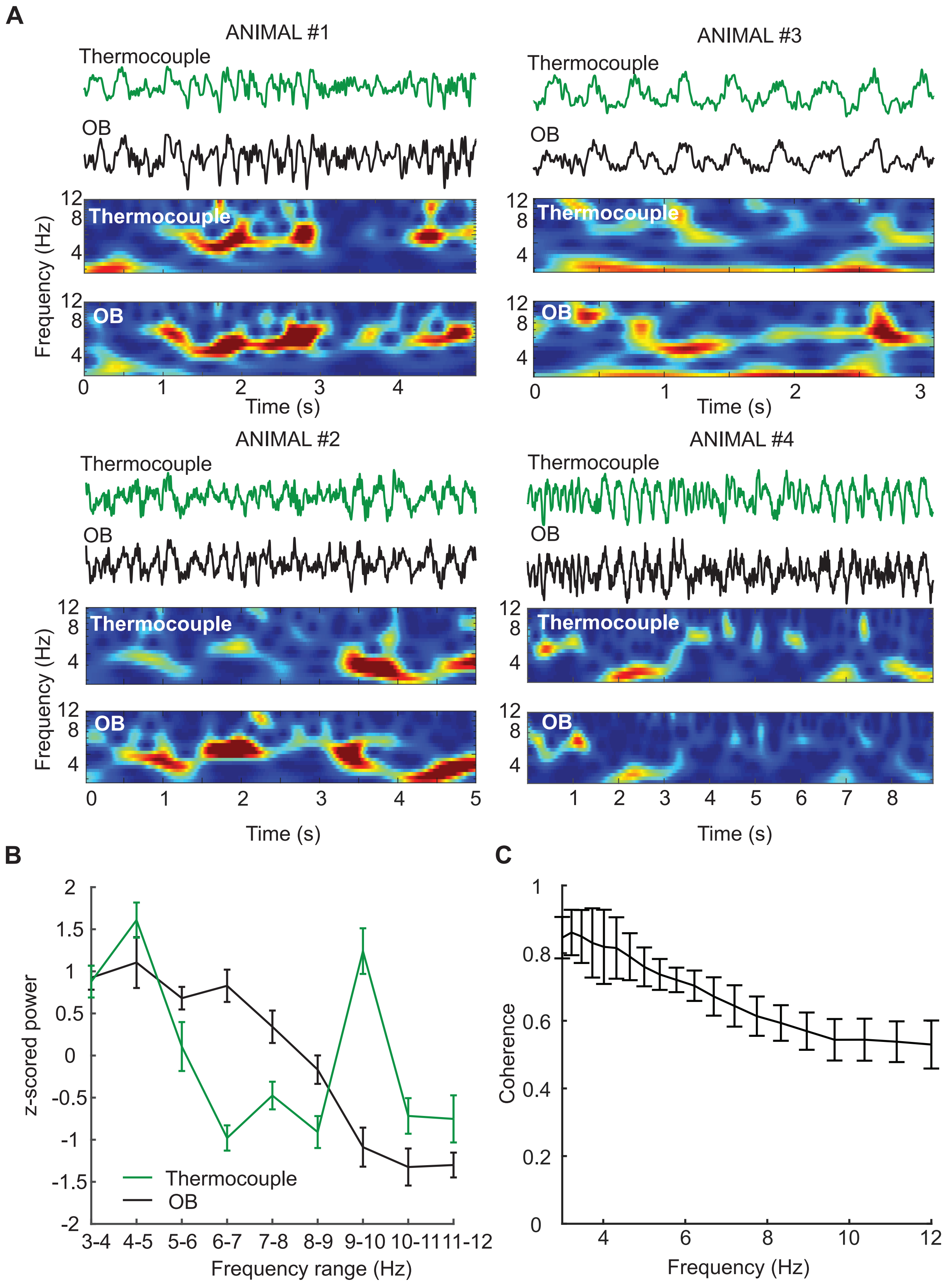

Figure S3. Respiration rhythm and OB oscillations were tightly coupled at all respiration frequencies. A. Example raw signals from a thermocouple measuring temperature in the nasal cavity (green) and an OB recording electrode (black) and the corresponding spectrograms from four animals. **B**. Z-scored power of thermocouple (green) and OB (black) are plotted as a function of frequency. Power of OB oscillations decreased at higher frequency ranges while thermocouple power did not show a consistent pattern. Lines indicate mean±std (n = 4 mice). **C**. Coherence between OB and thermocouple remained high across all frequencies. Black line indicates mean±std (n = 4 mice). These recordings confirm that OB oscillation frequency in the 3-12 Hz range can be used as a readout for respiration rate.

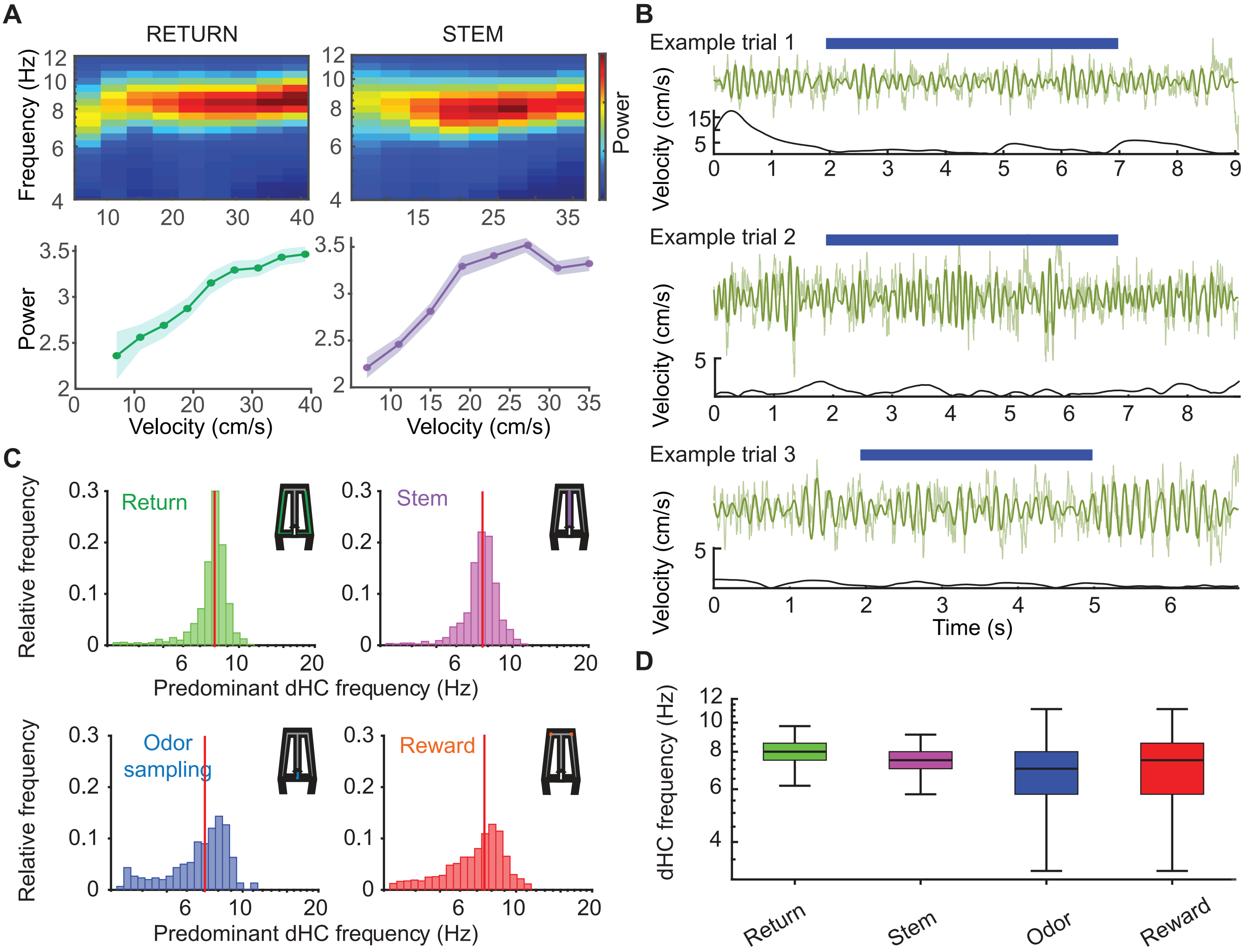

Figure S4. Movement-related and sensory-evoked theta oscillations in the dHC. A. (Top) Mean spectrograms during periods of running on the return arms and stem arm, displaying the distribution of power and frequency of movement-related theta oscillations across running speeds. (Bottom) Corresponding average power (±SEM) across running speeds. For each run through the return or the stem arm, the velocity of the mouse and dHC power were calculated, and the data were averaged across trials. **B**. Example raw (light green) and 3-12 Hz filtered (dark green) LFP traces of sensory-evoked theta oscillations in the dHC are shown in three different trials. Blue bars represent the time of odor sampling. Velocity traces accompanying each raw LFP trace show the velocity of the animal during the same time period. As expected, velocity was near zero while mice where holding their nose in the odor port. **C**. Distributions of predominant dHC LFP frequencies in each task phase. Vertical red line, median. **D**. Box plots of predominant dHC frequencies in each task phase. Data in C and D corresponds to **Figure 3B**, but more detailed distributions are shown here. See **Figure 3B** for statistics. Box plots: center line, median; bottom and top edges of the box, 25th and 75th percentiles; whiskers, most extreme data points not considered outliers.

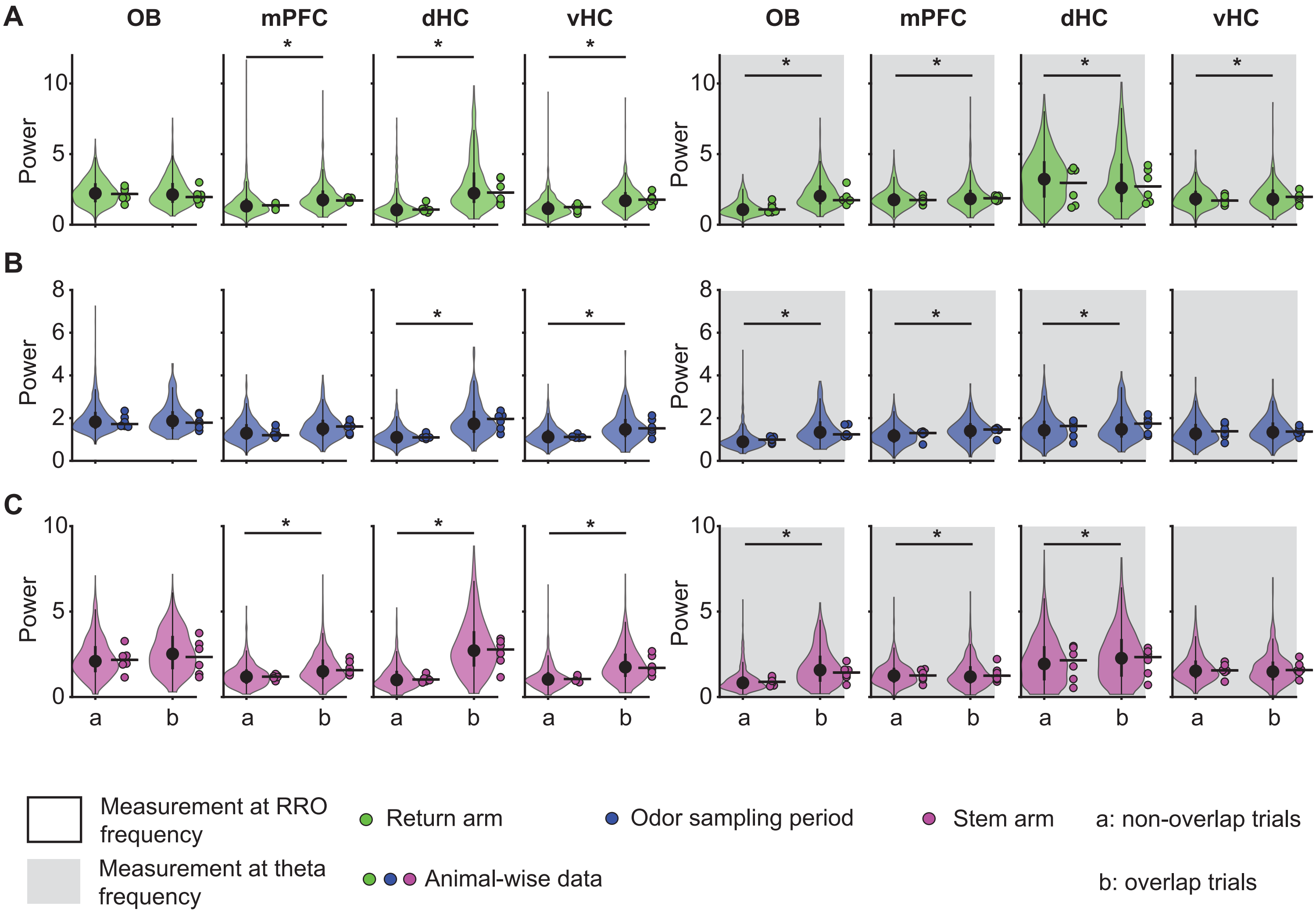

Figure S5. RRO and theta oscillation amplitude in non-overlap and overlap trials. Oscillation amplitude at RRO frequency was often higher in cortical areas in overlap trials compared to non-overlap trials. Oscillation amplitude at theta frequency did not change in a consistent direction in overlap compared to non-overlap trials. See Table S2 for detailed statistics.

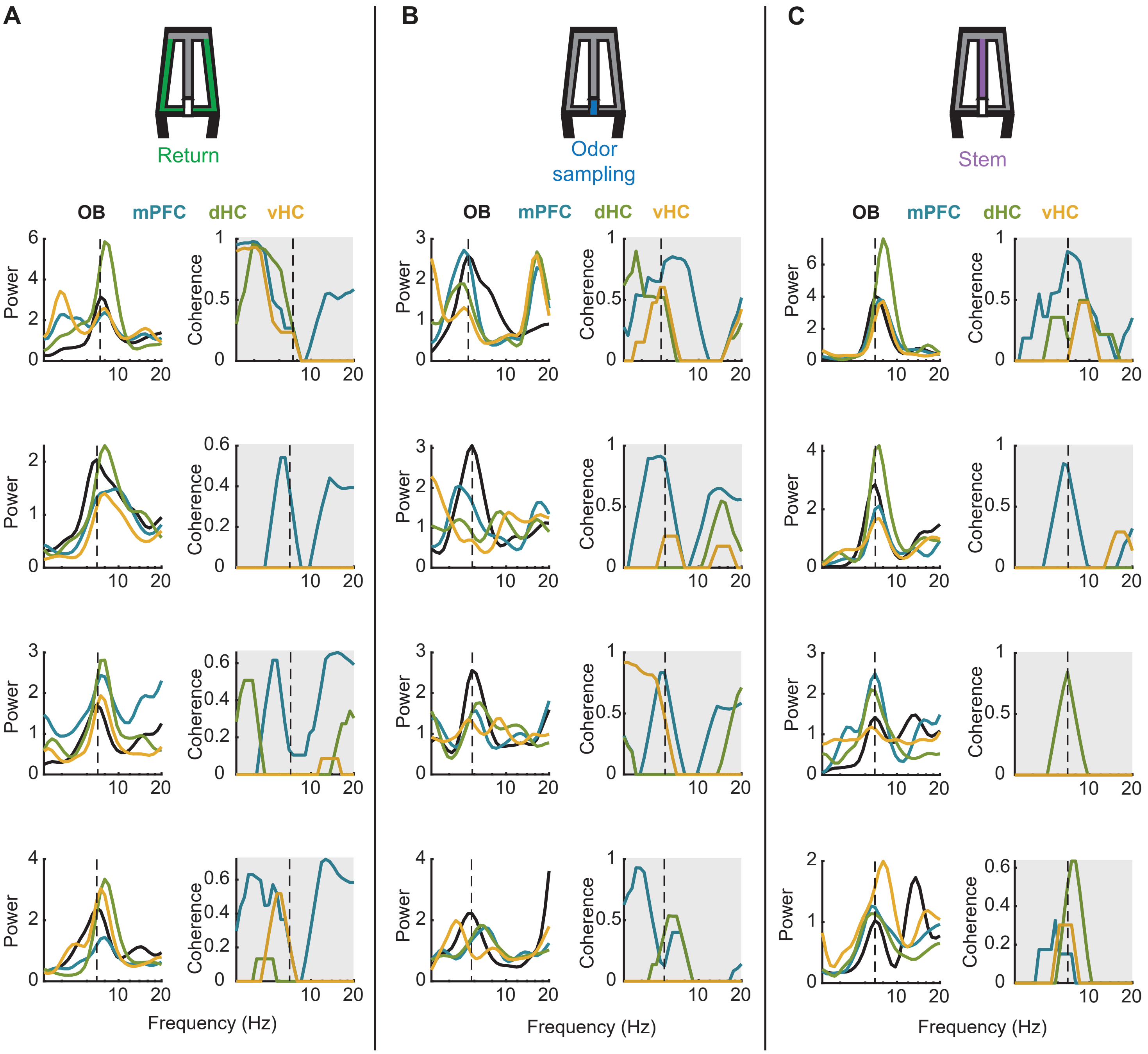

Figure S6. Example power and coherence spectra of trials with overlapping OB and theta frequency. Examples of time averaged power spectra (left) and coherence spectra (right) are shown for four trials in each maze zone (**A**, return arms; **B**, odor sampling period; **C**, stem arm) when OB and canonical theta frequencies were overlapping. Dotted lines indicate the frequency of the predominant OB oscillation in the respective trials. OB-mPFC (blue), OB-dHC (green) and OB-vHC (yellow) coherence was not always high at the frequency matching the predominant OB frequency. Moreover, only a subset of pairs of recording sites was significantly coherent at the predominant OB frequency. Coherence values that are not significantly different from chance are shown as zero at the respective frequency bin.

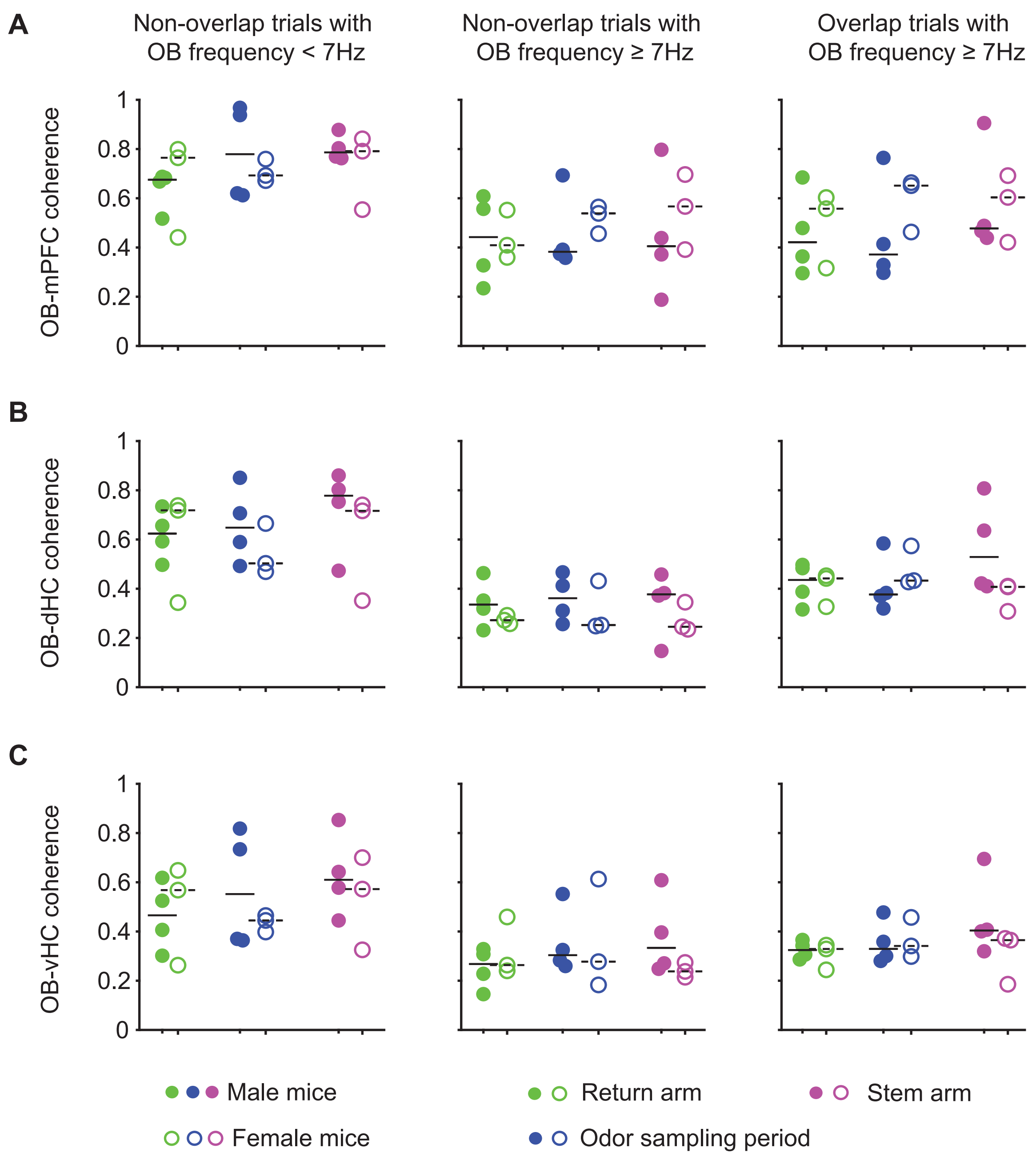

Figure S7. OB-mPFC, OB-dHC and OB-vHC coherence by sex. A. For each combination of maze zone (return arm, odor sampling period, stem arm) and frequency condition (Non-overlap < 7 Hz, Non-overlap ≥7 Hz, Overlap ≥7 Hz) OB-mPFC coherence values were compared between male and female mice. From left to right: chi-sq = 3.68, 0.41, 0.00, 0.18, 0.01, 0.02, 1.14, 4.86, 0.08; p = 0.05, 0.52, 1.00, 0.67, 0.94, 0.89, 0.29, 0.03, 0.78; Kruskal Wallis test. All p values n.s. after Holm-Bonferroni correction. **B**. As A, but for OB-dHC coherenece. From left to right: chi-sq = 2.23, 4.25, 0.32, 3.68, 0.41, 3.68, 0.08, 2.02, 1.46; p = 0.14, 0.04, 0.57, 0.06, 0.52, 0.06, 0.78, 0.16, 0.23; Kruskal Wallis test. All p values n.s. after Holm-Bonferroni correction. **C**. As A, but for OB-vHC coherenece. From left to right: chi-sq = 1.46, 0.85, 0.08, 0.13, 0.62, 6.19, 1.64, 0.32, 2.23; p = 0.23, 0.36, 0.78, 0.72, 0.43, 0.01, 0.2, 0.57, 0.14; Kruskal Wallis test. All p values n.s. after Holm-Bonferroni correction.

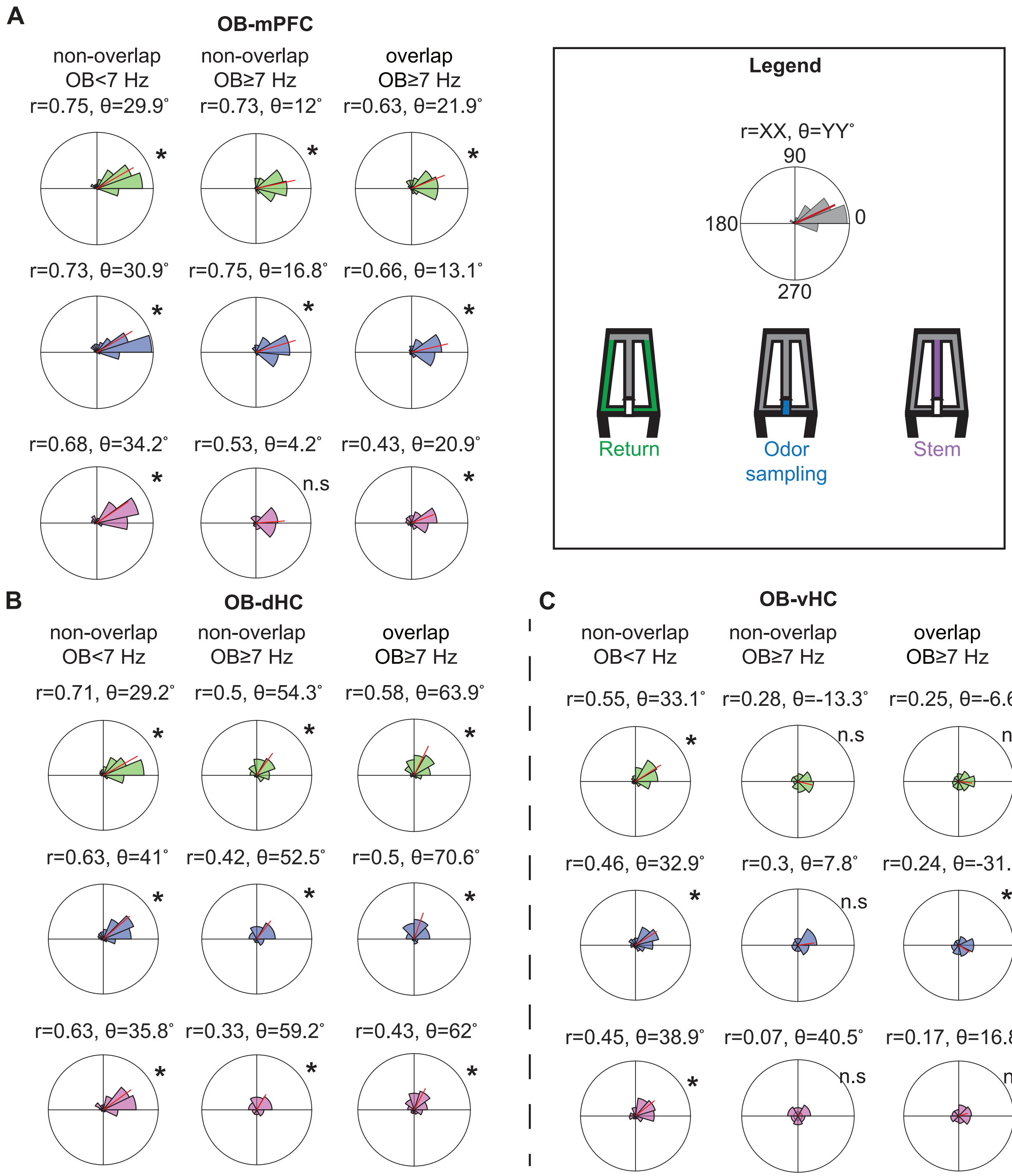

Figure S8. Phase differences between OB oscillations and oscillations in the mPFC, dHC and vHC during non-overlap trials with OB frequency <7 Hz, non-overlap trials with OB frequency ≥7 Hz and overlap trials with OB frequency ≥7 Hz. A. Phase differences between OB and mPFC oscillations were significantly non-zero except in the odor sampling period during non-overlap trials with OB frequencies ≥7 Hz. **B**. Phase differences between OB and dHC oscillations were significantly non-zero during all task phases and trial types. **C**. Phase differences between OB and vHC oscillations were significantly non-zero except in the return and stem arms when OB frequency was >7 Hz and except in the odor sampling period during non-overlap trials with OB frequencies ≥7 Hz. Polar plots show the distribution of phase differences for the three trial types in three task phases (top, return arm, middle, odor sampling , bottom, stem arm). The red line indicates the mean vector of the phase differences. The mean vector length (r) and mean phase difference (θ) are displayed above each polar plot. * and n.s., significant and non-significant phase differences; significant, $p < 0.05$.

Table S1. Number of overlap and non-overlap trials with OB frequency < 7 Hz and ≥7 Hz.

Return					
Animal	Total trials	non-overlap OB <7 Hz	overlap OB <7 Hz	non-overlap OB >=7 Hz	overlap OB >=7 Hz
Animal 1	169	12	1	56	99
Animal 2	175	47	6	32	89
Animal 3	175	2	0	63	103
Animal 4	175	143	7	6	9
Animal 5	168	149	4	4	9
Animal 6	173	28	8	43	71
Animal 7	172	94	8	20	50
	1207	475 (39.4%)	34 (2.8%)	224 (18.6 %)	430 (35.6 %)
**Odor sampling**					
Animal	Total trials	non-overlap OB <7 Hz	overlap OB <7 Hz	non-overlap OB >=7 Hz	overlap OB >=7 Hz
Animal 1	169	138	2	1	1
Animal 2	175	86	19	28	37
Animal 3	175	73	10	17	30
Animal 4	175	129	4	10	16
Animal 5	168	141	9	1	10
Animal 6	173	89	16	16	28
Animal 7	172	124	9	15	24
	1207	780 (64.6%)	69 (5.7%)	88 (7.3%)	146 (12.1%)
**Stem**					
Animal	Total trials	non-overlap OB <7 Hz	overlap OB <7 Hz	non-overlap OB >=7 Hz	overlap OB >=7 Hz
Animal 1	169	49	1	26	90
Animal 2	175	51	21	20	83
Animal 3	175	3	1	53	110
Animal 4	175	141	2	13	3
Animal 5	168	121	20	6	18
Animal 6	173	53	27	16	22
Animal 7	172	108	16	7	41
	1207	526 (43.6%)	88 (7.3%)	141 (11.7%)	367 (30.4%)

Table S2. Statistics for comparisons of oscillation power between non-overlap and overlap trials in Figure S5.

Power measured at RRO frequency				
**Maze zone**	**OB**	**mPFC**	**dHC**	**vHC**
**Return**	chi-sq = 0.33, p = 5.64e-01	chi-sq = 11.44, p = 7.21e-04	chi-sq = 27.00, p = 2.03e-07	chi-sq = 16.33, p = 5.31e-05
**Odor sampling**	chi-sq = 1.53, p = 2.16e-01	chi-sq = 3.00, p = 8.33e-02	chi-sq = 16.33, p = 5.31e-05	chi-sq = 7.41, p = 6.49e-03
**Stem**	chi-sq = 0.01, p = 9.34e-01	chi-sq = 17.69, p = 2.59e-05	chi-sq = 22.10, p = 2.59e-06	chi-sq = 17.69, p = 2.59e-05

Power measured at theta frequency				
**Maze zone**	**OB**	**mPFC**	**dHC**	**vHC**
**Return**	chi-sq = 25.31, p = 4.87e-07	chi-sq = 4.25, p = 3.92e-02	chi-sq = 5.72, p = 1.68e-02	chi-sq = 9.31, p = 2.28e-03
**Odor sampling**	chi-sq = 19.11, p = 1.23e-05	chi-sq = 7.41, p = 6.49e-03	chi-sq = 6.54, p = 1.06e-02	chi-sq = 1.15, p = 2.84e-01
**Stem**	chi-sq = 15.03, p = 1.06e-04	chi-sq = 1.97, p = 1.61e-01	chi-sq = 4.25, p = 3.92e-02	chi-sq = 0.55, p = 4.58e-01

Table S3. Statistical comparisons for the data in Figure 5.

Friedman tests with sessions as repeated measures, followed by two-sided Wilcoxon signed-rank tests as post-hoc test.

Comparisons between task phases
a - Return arms
b - Odor sampling
c - Stem arm

	Non overlap trials, OB frequency < 7Hz			Non overlap trials, OB frequency >= 7Hz			Overlap trials, OB frequency >= 7Hz		
**OB-mPFC**	Friedman - chi-sq=7.18; p=0.0275			Friedman - chi-sq=2.05; p=0.359			Friedman - chi-sq=9.62; p=0.0081		
	a vs b	a vs c	b vs c	a vs b	a vs c	b vs c	a vs b	a vs c	b vs c
Signedrank	42	39	133	100	54	64	95	56	71
zval	-2.55	-2.46	0.61	-0.19	-1.90	-1.53	-0.71	-2.07	-1.55
p	0.011	0.014	0.543	0.852	0.057	0.126	0.476	0.039	0.122
holm bonferroni corrected significance	sig.	sig.	n.s.	n.s.	n.s.	n.s.	n.s.	n.s.	n.s.
**OB-dHC**	Friedman - chi-sq=2.33; p=0.3125			Friedman - chi-sq=2.28; p=0.3197			Friedman - chi-sq=5.3; p=0.0706		
	a vs b	a vs c	b vs c	a vs b	a vs c	b vs c	a vs b	a vs c	b vs c
Signedrank	94	57	97	139	93	66	126	76	66
zval	-0.75	-1.79	-0.64	1.27	-0.45	-1.46	0.36	-1.37	-1.72
p	0.455	0.073	0.520	0.204	0.654	0.145	0.715	0.170	0.085
holm bonferroni corrected significance	n.s.	n.s.	n.s.	n.s.	n.s.	n.s.	n.s.	n.s.	n.s.
**OB-vHC**	Friedman - chi-sq=2.58; p=0.2748			Friedman - chi-sq=5.16; p=0.0759			Friedman - chi-sq=7.36; p=0.0252		
	a vs b	a vs c	b vs c	a vs b	a vs c	b vs c	a vs b	a vs c	b vs c
Signedrank	84	57	106	94	76	86	111	60	64
zval	-1.09	-1.79	-0.33	-0.41	-1.08	-0.71	-0.16	-1.93	-1.79
p	0.274	0.073	0.741	0.681	0.279	0.478	0.876	0.054	0.073
holm bonferroni corrected significance	n.s.	n.s.	n.s.	n.s.	n.s.	n.s.	n.s.	n.s.	n.s.

Comparisons between trial types
a - Non overlap trials with OB frequency < 7Hz
b - Non overlap trials with OB frequency >= 7Hz
c - Overlap trials with OB frequency >= 7Hz

	Return			Odor			Stem		
**OB-mPFC**	Friedman - chi-sq=20.29; p=3.9255e-5			Friedman - chi-sq=34.14; p=3.87e-8			Friedman - chi-sq=17.43; p=0.0002		
	a vs b	a vs c	b vs c	a vs b	a vs c	b vs c	a vs b	a vs c	b vs c
Signedrank	191	187	73	231	228	71	186	180	66
zval	2.62	2.49	-1.48	4.01	3.91	-1.27	2.45	2.24	-1.72
p	0.009	0.013	0.140	0.000	0.000	0.204	0.014	0.025	0.085
holm bonferroni corrected significance	sig.	sig.	n.s.	sig.	sig.	n.s.	sig.	sig.	n.s.
**OB-dHC**	Friedman - chi-sq=22.79; p=1.03e-5			Friedman - chi-sq=36.25; p=1.35e-8			Friedman - chi-sq=21.41; p=2.24e-5		
	a vs b	a vs c	b vs c	a vs b	a vs c	b vs c	a vs b	a vs c	b vs c
Signedrank	198	187	34	231	223	28	203	175	31
zval	2.87	2.49	-2.83	4.01	3.74	-2.87	3.04	2.07	-2.94
p	0.004	0.013	0.005	0.000	0.000	0.004	0.002	0.039	0.003
holm bonferroni corrected significance	sig.	sig.	sig.	sig.	sig.	sig.	sig.	sig.	sig.
**OB-vHC**	Friedman - chi-sq=15.9; p=0.0004			Friedman - chi-sq=27.14; p=1.28e-6			Friedman - chi-sq=16.03; p=0.0003		

	Return			Odor			Stem		
	a vs b	a vs c	b vs c	a vs b	a vs c	b vs c	a vs b	a vs c	b vs c
Signedrank	191	179	56	221	231	89	186	175	43
zval	2.62	2.21	-2.07	3.67	4.01	-0.60	2.45	2.07	-2.52
p	0.009	0.027	0.039	0.000	0.000	0.550	0.014	0.039	0.012
holm bonferroni corrected significance	sig.	n.s.	n.s.	sig.	sig.	n.s.	sig.	sig.	sig.

Comparison to shuffle									
	**Return**			**Odor**			**Stem**		
**OB-mPFC**	a	b	c	a	b	c	a	b	c
Friedman chi-sq	21.46	8.85	20.58	27	13.13	17.03	19.92	7.9	14.45
Friedman p	0.000	0.003	0.000	0.000	0.000	0.000	0.000	0.005	0.000
**OB-dHC**	a	b	c	a	b	c	a	b	c
Friedman chi-sq	21.45	8.85	19.11	27	3.6	15.67	14.36	1.34	8.85
Friedman p	0.000	0.003	0.000	0.000	0.058	0.000	0.000	0.247	0.003
**OB-vHC**	a	b	c	a	b	c	a	b	c
Friedman chi-sq	10.79	2.21	3	15.03	2.41	3.13	6.82	0.98	3.31
Friedman p	0.001	0.137	0.083	0.000	0.121	0.077	0.009	0.321	0.069

Table S4. Statistical comparisons for the data in Figure 6.

Friedman tests with sessions as repeated measures, followed by two-sided Wilcoxon signed-rank tests as post-hoc test.

Measurements at RRO frequency

Comparisons between task phases

a - Return arms
b - Odor sampling
c - Stem arm

	**Non overlap trials, OB frequency < 7Hz**			**Non overlap trials, OB frequency >= 7Hz**			**Overlap trials, OB frequency >= 7Hz**		
**mPFC-dHC**	Friedman - chi-sq=3.86; p=0.1455			Friedman - chi-sq=8.21; p=0.0165			Friedman - chi-sq=12.37; p=0.0021		
	a vs b	a vs c	b vs c	a vs b	a vs c	b vs c	a vs b	a vs c	b vs c
Signedrank	127	53	75	169	84	37	170	49	31
zval	0.40	-1.94	-1.41	2.39	-0.78	-2.54	1.89	-2.31	-2.94
p	0.689	0.052	0.159	0.017	0.433	0.011	0.058	0.021	0.003
holm bonferroni corrected significance	n.s.	n.s.	n.s.	sig.	n.s.	sig.	n.s.	sig.	sig.
**mPFC-vHC**	Friedman - chi-sq=2.58; p=0.2748			Friedman - chi-sq=3.6; p=0.1653			Friedman - chi-sq=10.39; p=0.0056		
	a vs b	a vs c	b vs c	a vs b	a vs c	b vs c	a vs b	a vs c	b vs c
Signedrank	134	94	97	163	124	44	212	104	43
zval	0.64	-0.41	-0.64	2.17	0.71	-2.28	3.35	-0.40	-2.52
p	0.520	0.681	0.520	0.030	0.478	0.023	0.001	0.689	0.012
holm bonferroni corrected significance	n.s.	n.s.	n.s.	n.s.	n.s.	n.s.	sig.	n.s.	sig.
**dHC-vHC**	Friedman - chi-sq=0.63; p=0.7299			Friedman - chi-sq=8.89; p=0.0117			Friedman - chi-sq=14.51; p=0.0007		
	a vs b	a vs c	b vs c	a vs b	a vs c	b vs c	a vs b	a vs c	b vs c
Signedrank	128	99	115	175	62	16	171	38	25
zval	0.43	-0.22	-0.02	2.61	-1.61	-3.32	1.93	-2.69	-3.15
p	0.664	0.823	0.986	0.009	0.108	0.001	0.054	0.01	0.002
holm bonferroni corrected significance	n.s.	n.s.	n.s.	sig.	n.s.	sig.	n.s.	sig.	sig.

Comparisons between trial types

a - Non overlap trials with OB frequency < 7Hz
b - Non overlap trials with OB frequency >= 7Hz
c - Overlap trials with OB frequency >= 7Hz

	**Return**			**Odor**			**Stem**		
**mPFC-dHC**	Friedman - chi-sq=12.56; p=0.0019			Friedman - chi-sq=18.89; p=7.91e-5			Friedman - chi-sq=10.34; p=0.0057		
	a vs b	a vs c	b vs c	a vs b	a vs c	b vs c	a vs b	a vs c	b vs c
Signedrank	174	76	24	220	145	24	162	85	36
zval	2.03	-1.37	-3.18	3.63	1.03	-3.02	1.62	-1.06	-2.76
p	0.042	0.170	0.001	0.000	0.305	0.002	0.106	0.289	0.006
holm bonferroni corrected significance	n.s.	n.s.	sig.	sig.	n.s.	sig.	n.s.	n.s.	sig.
**mPFC-vHC**	Friedman - chi-sq=16.03; p=0.0003			Friedman - chi-sq=3.18; p=0.2041			Friedman - chi-sq=5.58; p=0.0614		
	a vs b	a vs c	b vs c	a vs b	a vs c	b vs c	a vs b	a vs c	b vs c
Signedrank	103	29	23	185	119	55	122	67	54
zval	-0.43	-3.01	-3.22	2.42	0.12	-1.87	0.23	-1.69	-2.14
p	0.664	0.003	0.001	0.016	0.903	0.062	0.821	0.092	0.033
holm bonferroni corrected significance	n.s.	sig.	sig.	sig.	n.s.	n.s.	n.s.	n.s.	n.s.

dHC-vHC	Friedman - chi-sq=13.59; p=0.0011			Friedman - chi-sq=14.76; p=0.0006			Friedman - chi-sq=16.11; p=0.0003		
	a vs b	a vs c	b vs c	a vs b	a vs c	b vs c	a vs b	a vs c	b vs c
Signedrank	110	39	14	213	111	28	113	31	22
zval	-0.19	-2.66	-3.53	3.39	-0.16	-2.87	-0.09	-2.94	-3.25
p	0.848	0.008	0.000	0.001	0.876	0.004	0.931	0.003	0.001
holm bonferroni corrected significance	n.s.	sig.	sig.	sig.	n.s.	sig.	n.s.	sig.	sig.

Comparison to shuffle									
	Return			Odor			Stem		
**mPFC-dHC**	a	b	c	a	b	c	a	b	c
Friedman chi-sq	21.45	22.98	27.00	27.00	7.62	11.92	21.45	12.05	22.98
Friedman p	0.000	0.000	0.000	0.000	0.006	0.001	0.000	0.001	0.000
**mPFC-vHC**	a	b	c	a	b	c	a	b	c
Friedman chi-sq	21.45	22.98	27.00	23.68	10.74	18.45	18.45	13.22	15.74
Friedman p	0.000	0.000	0.000	0.000	0.001	0.000	0.000	0.000	0.000
**dHC-vHC**	a	b	c	a	b	c	a	b	c
Friedman chi-sq	21.45	22.98	22.10	27.00	2.41	21.45	5.96	13.22	18.47
Friedman p	0.000	0.000	0.000	0.000	0.121	0.000	0.015	0.000	0.000

Measurements at canonical theta frequency

Comparisons between task phases

a - Return arms
b - Odor sampling
c - Stem arm

	Non overlap trials, OB frequency < 7Hz			Non overlap trials, OB frequency >= 7Hz			Overlap trials, OB frequency >= 7Hz		
**mPFC-dHC**	Friedman - chi-sq=14.12; p=0.0009			Friedman - chi-sq=9.56; p=0.0084			Friedman - chi-sq=9.32; p=0.0095		
	a vs b	a vs c	b vs c	a vs b	a vs c	b vs c	a vs b	a vs c	b vs c
Signedrank	187	98	50	169	78	30	196	105	43
zval	2.49	-0.26	-2.28	2.39	-1.01	-2.80	2.80	-0.36	-2.52
p	0.013	0.794	0.023	0.017	0.313	0.005	0.005	0.715	0.012
holm bonferroni corrected significance	sig.	n.s.	sig.	sig.	n.s.	sig.	sig.	n.s.	sig.
**mPFC-vHC**	Friedman - chi-sq=14.03; p=0.0009			Friedman - chi-sq=4.7; p=0.0954			Friedman - chi-sq=13.13; p=0.0014		
	a vs b	a vs c	b vs c	a vs b	a vs c	b vs c	a vs b	a vs c	b vs c
Signedrank	179	106	43	159	115	60	211	102	50
zval	2.21	0.04	-2.52	2.02	0.37	-1.68	3.32	-0.47	-2.28
p	0.027	0.970	0.012	0.044	0.709	0.093	0.001	0.639	0.023
holm bonferroni corrected significance	n.s.	n.s.	sig.	n.s.	n.s.	n.s.	sig.	n.s.	sig.
**dHC-vHC**	Friedman - chi-sq=17.21; p=0.0002			Friedman - chi-sq=8.84; p=0.012			Friedman - chi-sq=13.31; p=0.0013		
	a vs b	a vs c	b vs c	a vs b	a vs c	b vs c	a vs b	a vs c	b vs c
Signedrank	190	91	41	181	95	21	205	86	34
zval	2.59	-0.52	-2.59	2.84	-0.37	-3.14	3.11	-1.03	-2.83
p	0.010	0.601	0.010	0.005	0.709	0.002	0.002	0.305	0.005
holm bonferroni corrected significance	sig.	n.s.	sig.	sig.	n.s.	sig.	sig.	n.s.	sig.

Comparisons between trial types

a - Non overlap trials with OB frequency < 7Hz

b - Non overlap trials with OB frequency >= 7Hz

c - Overlap trials with OB frequency >= 7Hz

	Return			Odor			Stem		
**mPFC-dHC**	Friedman - chi-sq=4.12; p=0.1275			Friedman - chi-sq=0.27; p=0.8736			Friedman - chi-sq=1.92; p=0.3829		
	a vs b	a vs c	b vs c	a vs b	a vs c	b vs c	a vs b	a vs c	b vs c
Signedrank	132	65	51	142	117	90	97	90	115
zval	0.57	-1.76	-2.24	0.92	0.05	-0.56	-0.64	-0.89	-0.02
p	0.566	0.079	0.025	0.357	0.958	0.575	0.520	0.375	0.986
holm bonferroni corrected significance	n.s.	n.s.	n.s.	n.s.	n.s.	n.s.	n.s.	n.s.	n.s.
**mPFC-vHC**	Friedman - chi-sq=3.71; p=0.1563			Friedman - chi-sq=2.25; p=0.3244			Friedman - chi-sq=0.72; p=0.696		
	a vs b	a vs c	b vs c	a vs b	a vs c	b vs c	a vs b	a vs c	b vs c
Signedrank	110	63	78	120	67	93	110	94	85
zval	-0.19	-1.82	-1.30	0.16	-1.69	-0.45	-0.19	-0.75	-1.06
p	0.848	0.068	0.192	0.876	0.092	0.654	0.848	0.455	0.289
holm bonferroni corrected significance	n.s.	n.s.	n.s.	n.s.	n.s.	n.s.	n.s.	n.s.	n.s.
**dHC-vHC**	Friedman - chi-sq=0.93; p=0.6287			Friedman - chi-sq=0.73; p=0.693			Friedman - chi-sq=0.55; p=0.7608		
	a vs b	a vs c	b vs c	a vs b	a vs c	b vs c	a vs b	a vs c	b vs c
Signedrank	143	105	85	129	110	93	122	97	77
zval	0.96	-0.36	-1.06	0.47	-0.19	-0.45	0.23	-0.64	-1.34
p	0.339	0.715	0.289	0.639	0.848	0.654	0.821	0.520	0.181
holm bonferroni corrected significance	n.s.	n.s.	n.s.	n.s.	n.s.	n.s.	n.s.	n.s.	n.s.

Comparison to shuffle

	Return			Odor			Stem		
**mPFC-dHC**	a	b	c	a	b	c	a	b	c
Friedman chi-sq	21.45	22.98	27.00	23.68	8.60	8.69	21.45	18.47	17.08
Friedman p	0.000	0.000	0.000	0.000	0.003	0.003	0.000	0.000	0.000
**mPFC-vHC**	a	b	c	a	b	c	a	b	c
Friedman chi-sq	21.45	22.98	27.00	22.10	13.13	21.45	19.92	9.86	19.92
Friedman p	0.000	0.000	0.000	0.000	0.000	0.000	0.000	0.002	0.000
**dHC-vHC**	a	b	c	a	b	c	a	b	c
Friedman chi-sq	18.45	22.98	27.00	17.69	9.64	9.71	15.67	12.05	18.47
Friedman p	0.000	0.000	0.000	0.000	0.002	0.002	0.000	0.001	0.000
